# Unveiling covert disownership after stroke: a neuropsychological and neural approach

**DOI:** 10.1093/braincomms/fcaf217

**Published:** 2025-06-04

**Authors:** Eugénie Cataldo, Eda Tipura, Corrado Corradi-Dell’Acqua, Thomas Martin, Fabien Albert, Frédéric Assal, Patrik Vuilleumier, Roberta Ronchi

**Affiliations:** Department of Basic Neurosciences, Faculty of Medicine, University of Geneva, Geneva CH-1211, Switzerland; Department of Clinical Neurosciences, Geneva University Hospitals, Geneva CH-1211, Switzerland; Department of Basic Neurosciences, Faculty of Medicine, University of Geneva, Geneva CH-1211, Switzerland; Department of Clinical Neurosciences, Geneva University Hospitals, Geneva CH-1211, Switzerland; Faculty of Psychology and Educational Sciences, University of Geneva, Geneva CH-1211, Switzerland; Center for Mind/Brain Sciences (CIMeC), University of Trento, Rovereto 38068, Italy; Department of Clinical Neurosciences, Geneva University Hospitals, Geneva CH-1211, Switzerland; Department of Clinical Neurosciences, Geneva University Hospitals, Geneva CH-1211, Switzerland; Department of Clinical Neurosciences, Geneva University Hospitals, Geneva CH-1211, Switzerland; Department of Clinical Neurosciences, Faculty of Medicine, University of Geneva, Geneva CH-1211, Switzerland; Department of Basic Neurosciences, Faculty of Medicine, University of Geneva, Geneva CH-1211, Switzerland; Department of Clinical Neurosciences, Geneva University Hospitals, Geneva CH-1211, Switzerland; Department of Basic Neurosciences, Faculty of Medicine, University of Geneva, Geneva CH-1211, Switzerland; Department of Clinical Neurosciences, Geneva University Hospitals, Geneva CH-1211, Switzerland; Department of Clinical Neurosciences, Faculty of Medicine, University of Geneva, Geneva CH-1211, Switzerland

**Keywords:** bodily self-consciousness, lack of ownership, brain lesion analyses, structural disconnection, neuropsychology

## Abstract

Self-awareness can be impaired in different forms, including bodily features, following brain lesions. Such as other complex symptoms, these disorders seem not being localized in one brain area but may occur following the impairment of different parts of a network. One of the most disrupting body awareness disorder for people's functioning is the feeling that one or more body parts do not belong to the person anymore, the so-called ‘body part disownership’. This symptom can be undetected, with recent findings suggesting that subtle signs of body disownership are revealed using an assessment with a non-verbal response, instead of a verbal interview. In the present study, by exploiting a large clinical dataset and state-of-the art analyses of lesion-induced disconnectivity, we have investigated this newly detected entity, called covert disownership, in the early phase post-stroke. 105 hospitalized stroke patients and 55 healthy controls were recruited over 2 years. Patients underwent a neurological and cognitive evaluation, including various measures of body ownership. We also assessed upper limb functions, using routine occupational therapy measures such as hand dexterity, strength, sensitivity and proprioception. To unveil its neural correlates, we ran innovative and robust region- and network-based lesion analyses. Our results indicate that about 30% of our sample exhibited covert disownership, involving either hands, arms, legs or face portions contralateral to the lesion, which affected the right- or the left-hemisphere. Lesion analyses confirmed the key role of structures such as the right insula, and basal ganglia, for upper limb ownership. Network-based structural connectivity data highlighted disconnections between temporo-occipital and parietal bilateral networks associated with disownership for the upper limb, as well as bilateral disconnections between fronto-basal and occipital parcels. Damage affecting the left superior longitudinal fasciculus was also linked to the right hand disownership. Altogether, we shed new light on the neural interconnections that, when perturbed, lead to body awareness disorders. Large-scale bilateral disconnections at the level of temporo-parietal and fronto-occipital networks explain covert disownership, with specific pathways as a function of the body part involved. We demonstrate that in the early phase of a brain damage this disorder may be underestimated but still affect patients’ self-perception. This underscores the importance of sensitive tools to overcome the limitations of standard clinical examination, as well as of modelling brain damage in terms of networks rather than focal lesions. A better understanding of post-stroke disownership disorders may improve rehabilitation programs and predict optimal clinical outcome.

## Teaser

After brain damage, patients can experience the lack of ownership of body parts. By exploiting a large dataset and state-of-the-art analyses, Cataldo *et al*. demonstrate that subtle forms of disownership are undetected post-stroke, and suggest the role of complex bilateral brain networks in the construction of body awareness in humans.

## Graphical Abstract

**Graphical Abstract:**
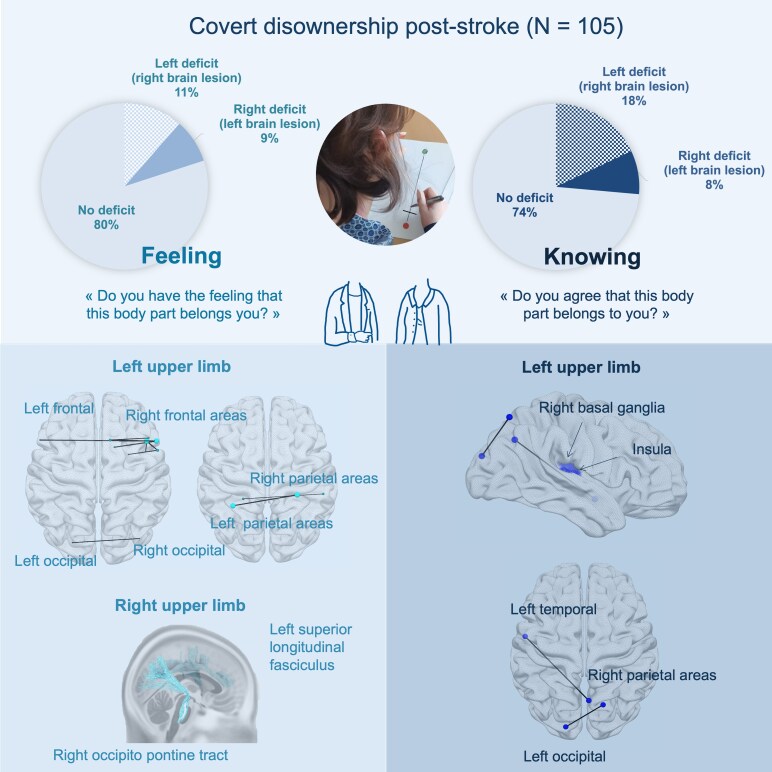

## Introduction

Our body, central to human perceptions, actions and cognition is the object we know the best.^1^ It is critical for survival, and highly salient for the individual's identity and well-being.^2,3^ Based on multisensory integration, in normal condition, we never call into question the feeling of ownership.^4,5^ Yet, the link between body and self can be compromised by neurological conditions.^5,6^ Brain-damaged patients can experience that one or more body parts do not belong to them anymore. This neuropsychological symptom is known as ‘body part disownership’, and has been more frequently found following a right hemisphere stroke.^7^ Although many studies have reported this pathological manifestation, we are far from a full comprehension of it. Recent research suggests that a failure to update multisensory signals might be responsible for such disorder, possibly caused by an alteration of fronto-temporo-parietal and insular structures.^8,9^ More precisely, one key assumption is that disownership reflect a deficit in a dynamic interaction between the body and the external space.^10-12^

This manifestation is usually detected with a verbal interview and called ‘disturbed sensation of ownership’^13^ or overt disownership (OD).^14^ Interestingly, Romano and Maravita^12^ argued that this symptom could also manifest at a more implicit level, and recent data suggest that OD is not the only manifestation within this spectrum. By asking for a non-verbal response, using a visual analogue scale (VAS), a more subtle form has been identified, called covert disownership^14^ (CD). This taxonomy has been proposed to take into account a disregarded sense of ownership in patients, declared neither spontaneously nor during an interview. By consequence, patients considered as unaffected may nonetheless have a perturbed bodily representation, which could negatively affect their self-perception. These first observations highlighted the importance of detecting this disorder, as a disown body part may be ‘disregarded’ or ‘rejected’: many reports in the literature^15-17^ describe the difficulty of living in a body that is perceived as ‘not me’, and even mild manifestations may trigger important consequences.

In the present study, we aimed at providing unprecedented evidence about this newly identified disorder in the early phase post-stroke and shed light on its neural correlates exploiting a large dataset. As main hypotheses, we predict that body disownership in its covert form is underestimated in the early phase post-stroke, and we expect to find a prevalence equal or even higher than in the sub-acute to chronic phase (about 25% in the previous study^6,14^), affecting selectively different body parts. Moreover, we hypothesize that patients with left brain damage may also present with higher degree of disownership than reported in the literature, explored until now using a verbal assessment,^12^ and this can hint at the left hemisphere's involvement in this disorder. Unveiling the clinical profile of patients, we predict that the (dys)functionality of the affected limb may play a critical role, as it has been shown for patients with distorted body representation following stroke or chronic pain.^18,19^ Finally, taking advantage of the extent and heterogeneity of our population, as complex cognitive deficits may be better explained by widespread patterns of abnormal connectivity, we hypothesize that CD relies on a network involving intra- (see studies exploring disownership predominantly in right-brain damaged patients^9,20^) and inter-hemispheric areas (see body ownership in healthy participants^21^), possibly involving connections including the basal ganglia and insula,^22,23^ but also fronto-parietal connections.^9,24^ Consequently, we planned to identify the neural features of disturbed body ownership by combining state-of-the-art lesion-based symptom mapping with connectivity analyses and applying leading-edge robust methodological procedures.

## Materials and methods

### Participants

In this prospective group study, we recruited 105 first-event stroke patients (64 males; mean age: 62.8 years old; SD: 16; range: 26–88) in the stroke unit of the Neurology service of Geneva University Hospitals (Switzerland), during their hospitalization (1–17 days after the stroke onset). This sample size was chosen to obtain a good estimation of the prevalence of the disorder, including a comparable number of patients with left- and right-brain lesions, and maximizing the requirements for reliable lesion analyses.^25^ Lesion site was assessed by magnetic resonance imaging (MRI) scans and included unilateral right (*N* = 55) and left (*N* = 45) lesions, as well as five bilateral strokes. Stroke aetiologies was ischaemic in 74 patients (37 right, 35 left, and 2 bilateral lesions), ischaemic with haemorrhagic transformation in 25 patients (14 right, 9 left and 2 bilateral lesions), haemorrhagic in 6 patients (4 right, 1 left and 1 with bilateral lesions). None of the participants had history or evidence of previous neurological (including traumatic brain injury) and psychiatric diseases, global cognitive deficits, or too severe aphasic deficits (poor tasks’ comprehension).

Fifty-five right-handed unimpaired participants (25 males; mean age: 63.2 years old; SD: 13.4; range: 32–86), neurologically age- (*t* = 0.016, *P* = 0.493) and gender-matched (*t* = −1.868, *P* = 0.064) with patients took part in the study as a control (C) group. Participants were recruited over 2 years (October 2021-June 2023). The study was approved by the Local Ethical Committee; participants’ written informed consent was given according to the Declaration of Helsinki.

### Clinical assessment

#### Neurological and neuropsychological evaluation

All patients underwent a neurological evaluation (motor, somato-sensory, visual and auditory) as part of the standard procedure in the stroke unit, using the National Institutes of Health Stroke Scale^26^ (NIHSS). In addition, cognitive deficits were assessed by the trained psychologists. A screening for global cognitive efficiency, global executive functioning and linguistic deficits was done^27-29^ (see Supplementary Materials: Supplementary Information). Then, an exhaustive evaluation of spatial attention processing was performed: unilateral spatial neglect (USN) affecting the visuo-exploratory, perceptual, representational components, as well as egocentric (i.e. based on the mid-sagittal plan of the patient) and allocentric (i.e. based on the contralesional part of the object, independently from the position of the body) deficits were assessed. As some stroke patients may present with altitudinal neglect (i.e. ignoring the upper or lower part of the space), which can affect our measure of CD through the vertical VAS, a vertical line bisection was administered. Personal neglect was also assessed by two tasks including a body part reaching with eyes open, and a body exploration test with eyes closed (see Supplementary Information for a detailed description of spatial and personal neglect tasks). We also examined the presence of awareness of the neurological disorders and subsequent deficits (i.e. anosognosia^30,31^). Finally, handedness (Edinburgh Handedness Inventory),^32^ level of anxiety and depression (Hospital Anxiety and Depression scale)^33^ and optimism traits (Life Orientation Test-Revised)^34^ were assessed through questionnaires to characterize the profile of patients in the context of a recent hospitalization.^35,36^

#### Upper limb functional assessment

We included some measures currently used in the clinical occupational therapy evaluation, to explore any potential association of affected upper limb functionality and CD. Manual dexterity was assessed using the Nine Hole Peg Test,^37^ hand grip strength was assessed with the Jamar hand dynamometer,^38^ finger sensibility was tested using the Two-points discrimination test^39^ to discern between one or two tactile stimulations delivered. Lastly, we assessed proprioception (left and right fingers, hands and feet) using an adaptation of the Distal Proprioception Task^40,41^ (see Supplementary Information for a detailed description of the tasks).

#### Disownership

CD was assessed for four body parts (palm of the hand, forearm, leg and face at the level of the cheek) on the left and right hemi-bodies, with the ipsilesional side providing a control condition, using a vertical VAS of 21 cm, and with two methodologies to better characterize the disorder: (i) as previously tested,^14^ with the patients indicating on the visual scale the agreement that a body part belongs to them; (ii) with the patients indicating the level of agreement that a body part felt like their own. With these two assessments, we wanted to distinguish between the (cognitive) judgment of ownership (Knowing form) and the Feeling form of body ownership, previously described in the literature.^1,42^ The score of the VAS ranged between zero (no knowledge/feeling of ownership) and 21 (total knowledge/feeling of ownership).

Moreover, we also evaluated the presence of OD, using a standardized interview, in which the examiner pointed at each body part asking ‘whom is this…. hand?’: the score ranges between zero and four, with four representing immediate recognition and no sign of OD, and zero representing spontaneous misattribution of ownership to another person.

The order of body parts, body sides, and type of evaluation (OD or CD) was randomized across participants. Within the CD assessment, the order of the two questions was kept fixed, namely: first, the ‘knowing’, second, the ‘feeling’ of ownership.

### Procedure

#### Selection of patients

A trained psychologist performed the neuropsychological and disownership assessments. The occupational therapist collected the measures of upper limb functionality. Of the 119 patients initially included, the final sample consisted of 105 patients.

#### Disownership

For CD, we computed a cut-off of contralesional body disownership for each body part, as the difference between contralesional versus ipsilesional scores. Based on the performance of healthy participants in the VAS task, in line with a previous study,^14^ we considered a defective ownership score a value greater than three standard deviations (SD) from (i) the healthy controls’ average scores, plus (ii) a lateralized left-right (L-R) difference from the heathy controls, for each body part (see Supplementary Table 1). We considered this method conservative to correctly classify contralesional deficits, and based on the distribution of data (with little variation of the scores in healthy controls). Patients were classified as showing CD if both scores were defective for body parts contralateral to the brain lesion, to guarantee a correct criterium of classification of unilateral/contralesional impairment.^7^ This procedure was applied for both the knowledge and the feeling of disownership.

Patients were classified as showing OD if they failed to acknowledge ownership for one or more controlesional body parts (score between zero and three) in the standard interview, based on a well-validated procedure.^14^

In OD assessment, all healthy participants evaluated body parts with a score of four (no impairment). Supplementary Table 1 shows the cut-off scores of OD and CD for each body part. Table 1 reports the demographic description of patients with OD, covert impaired knowledge of ownership (the body part belongs) and/or covert impaired feeling of ownership (the body part is felt).

**Table 1:** Demographical information of the 32 stroke patients with OD, knowing CD and/or feeling CD

Patient	OD	Knowing CD	Feeling CD	Gender^a^	Age	Education^b^	Time since stroke^c^ (days)	Take over delay^d^ (hours)	Aetiology^e^/Lesion site^f^/Side^g^
P001		1	n/a	M	72	2	17	575.5	I-H/ Silvian/R
P011		1	n/a	F	82	2	3.5	12.3	I/Silvian/R
P013		1	n/a	M	62	1	6.5	4.3	H/Silvian/R
P014	1	1	n/a	M	51	2	16	0.8	I-H/F-T-P/B
P018		1	n/a	M	77	3	6	0.7	I-H/Silvian/R
P022	1	1	n/a	M	88	2	7	2	H-I/th-F/B
P026		1	n/a	M	68	3	3	0.6	I/Silvian/R
P027		1	n/a	M	66	2	5	18.3	I/WM/R
P028	1	1	n/a	F	69	2	2	52.6	I/WM/L
P030		1	n/a	M	63	2	3	2.5	I/F/R
P033		1	n/a	F	52	3	2	11.4	I-H/O-P-In/L
P034		1	n/a	F	86	2	3	1	I/T-O-th/L
P038		1	n/a	F	37	2	9	1.9	H/F/R
P042			1	F	60	3	1	2.9	I-H/In-th/R
P043		1	1	M	83	2	3	25.7	I/bg/L
P046			1	M	58	3	3	1.7	I-H/In-P/L
P049		1	1	M	72	3	1	5.3	I/F-P/R
P053		1		M	75	3	3	47.9	I/WM-F-P/R
P061			1	M	66	2	3	2	I/Silvian/L
P068		1		M	53	3	7	115.3	I/WM/R
P073			I	F	73	1	5	5.4	I/Silvian/L
P079		1	1	F	60	2	2	4.7	I-H/Silvian/R
P081		1	1	M	37	1	6.5	2.9	I/Silvian/L
P086		1	1	F	84	1	4	14.3	I/F/L
P088		1	1	F	88	2	3	14.7	I/th-T-O/B
P092		1		F	56	1	4	6.2	I/T-P/R
P103		1	1	M	78	3	2.5	1.5	H/T/R
P106	1	1	1	M	59	2	8	32.2	I/WM-Silvian/R
P110		1	1	F	52	2	1	26.7	I/WM-ic/R
P112			1	M	52	1	2	3.6	I/F-T/R
P117		1		F	69	2	2	4.6	I-H/Silvian/R
P118		1		F	60	3	2	0.9	I/th/L

^a^M/F = male/female.

^b^1 = mandatory school level (up to 12 year); 2 = professional education (up to 18 year); 3 = high level education (university degree).

^c^Time since stroke = days since stroke onset.

^d^Take over delay (hours) = [arrival time at the emergency department—(time of the latest evidence of good health—first detected symptoms)/2].

^e^I/H = ischaemic/haemorrhagic lesion.

^f^F/P/T/O = frontal/parietal/temporal/occipital lesion; In = insula; th = thalamus; bg = basal ganglia; ic = internal capsule; WM = white matter fibres.

^g^R/L/B = right/left/bilateral; n/a = not assessed/missing data.

#### Associated deficits

Table 2 reports the clinical scores of patients with disownership. Patients were classified as showing unilateral spatial and/or personal neglect if they presented with defective, spatially lateralized (left or right), scores in at least one task, based on normative data^43-48^ (see Supplementary Information).

**Table 2:** Neurological and neuropsychological assessment of the 32 patients with OD, knowing CD and/or feeling CD

		SS			ANN for deficits^c^				Spatial neglect^d^		Personal neglect		Hand functionality		Proprioception	Anxiety	Depression		Optimism
Patient	M	NIHSS^a^	V	ANN for illness^b^	M	SS	V	5 cm	20 cm	CoC	Fluff^e^	Signs of neglect^f^	NHPT^g^	Jamar^h^	2 points^i^	DPT^j^	HAD^k^		LOT-r^l^
P001	3	2	0	-	1	3	n/a	1.5	17^m^	0.892^m^	−73.3^m^	+	[16.7]	[−39.7]	n/a	n/a	7	2	20
P011	0	0	0	-	n/a	n/a	n/a	2.5	15.5^m^	0.012	2.2	+	n/a	−16.3	0	10	11	12	18
P013	1	2	1	+	1	1	3	9^m^	22.5^m^	0.924^m^	n/a	+	[13.5]	[−32]	n/a	n/a	11	7	18
P014	2	2	2	-	0	1	1	0	6	0.382^m^	−71.1^m^	+	[14]	[−45]	n/a	5	9	6	19
P018	0	0	3	-	n/a	n/a	3	−5.5^m^	33^m^	0.996^m^	0	+	n/a	−2	0	8.83	6	7	6
P022	0	0	0	-	n/a	n/a	n/a	2	0	n/a	−13.3	+	−154	0.67	3	8.83	7	3	17
P026	0	0	0	-	n/a	n/a	n/a	1	5	0	0	+	1	−2	1	10	4	0	22
P027	0	0	0	-	n/a	n/a	n/a	−0.5	1.5	0.008	0	-	24	−9.33	1	10	5	3	15
P028	0	0	n/a	+	n/a	n/a	n/a	1.5	3.5	0.005	−40^m^	+	−4	2	−2	10	10	8	10
P030	0	0	0	-	n/a	n/a	n/a	1	1	0	−6.7	-	0.9	4	0	10	9	6	11
P033	0	0	0	-	n/a	n/a	n/a	−0.5	−1.5	−0.006	−40^m^	+	3.84	−1.4	0	9	16	4	22
P034	0	0	1	-	n/a	n/a	1	n/a	−68^m^	−0.653^m^	−40^m^	+	−2	2	−3	10	4	11	20
P038	0	0	0	-	n/a	n/a	n/a	3^m^	7.5^m^	−0.289^m^	−48.9^m^	+	16	−9	0	10	13	4	14
P042	0	0	0	-	n/a	n/a	n/a	−1	1	0.015	0	-	1.33	−2	−2	10	7	1	23
P043	0	0	0	-	n/a	n/a	n/a	0.5	4.5	−0.029	4.4	-	4.5	8	−1	10	7	3	19
P046	0	1	0	-	n/a	0	n/a	−0.5	1	0	−40^m^	+	−8	6	102	10	9	5	17
P049	0	0	0	-	n/a	n/a	n/a	−0.5	−1	0	−37.8^m^	+	0.61	2	0	10	7	2	15
P053	0	1	0	-	n/a	0	n/a	1.5	5.5	−0.031	8.9	+	8	0	1	10	0	0	20
P061	0	1	0	-	n/a	0	n/a	−2	−11^m^	−0.037	0	+	−5	10.7	−2	10	1	6	16
P068	0	1	0	-	n/a	0	n/a	0	3.5	0.042	2.2	+	n/a	n/a	1	10	13	6	12
P073	0	0	0	-	n/a	n/a	n/a	−1.5	0	0	−6.7	-	14.5	−16	15	10	3	8	17
P079	1	2	1	-	0	3	2	2.5^m^	7.5^m^	−0.049	−40^m^	+	−44	−14	n/a	9.17	8	4	16
P081	2	1	3	-	0	0	1	0.5	8^m^	0.002	−6.7	+	[−11.1]	[37.3]	−6	5	9	5	18
P086	0	0	0	-	n/a	n/a	n/a	−2.5	6.5	0	4.4	+	n/a	n/a	−2	9.33	4	0	22
P088	0	0	0	-	n/a	n/a	n/a	−3^m^	−3.5	n/a	13.3	+	20	−1	0	10	7	7	12
P092	0	0	0	-	n/a	n/a	n/a	0	−5	−0.005	−11.1	+	0.79	3	0	10	10	3	18
P103	1	2	0	-	0	1	3	−1	24.5^m^	0.191^m^	−66.7^m^	+	[16.7]	−18.7	n/a	5	10	7	9
P106	2	2	0	-	0	0	0	11.5^m^	46^m^	0.637^m^	−86.7^m^	+	[14]	[−34.3]	n/a	5	9	8	24
P110	0	0	0	-	n/a	n/a	n/a	3^m^	1	0	0	+	n/a	−4.6	1	10	6	2	19
P112	0	0	0	-	n/a	n/a	n/a	0.5	0.5	0	0	+	0.4	6.34	−1	10	8	0	20
P117	1	0	0	-	0	n/a	n/a	1.5	7.5^m^	−0.010	−11.1	+	3.69	−3	0	10	5	2	14
P118	0	0	0	-	n/a	n/a	n/a	1	2.5	0.012	0	-	8	0	0	10	9	6	15

^a^M/SS/V = motor/somatosensory/visual field deficits (scores extracted from the NIHSS: score ranging from 0 (no neurological deficit) to 42 (maximal neurological deficit).

^b^Anosognosia for illness + = patients showing signs of anosognosia for illness following Cutting's questionnaire.^1^

^c^Anosognosia for neurological and cognitive deficits = Bisiach's score^2^ from 0 to 3 (M/SS/V = motor/somatosensory/visual field deficits).

^d^5 and 20 cm = BEN line bisection: scores indicate the deviation in mm from the objective mid-point of the line; CoC = centre of cancellation (Apples test): positive CoC indicate a right-ward bias, negative scores a left-ward bias.

^e^PN score = personal neglect score (Fluff test), computed following the improved scoring system to account for different degrees of contralesional and ipsilesional personal neglect.^3^

^f^Neglect + = patients with defective scores in at least one task (spatial or personal).

^g^NHPT = Nine-hole peg test (this score indicates the difference in seconds of performing the task with the left-right hand).

^h^Jamar = this score indicates the difference in kilogram of performing the task with the left-right hand.

^i^2 points = this score indicates the difference in mm of performing the task with the left-right hand (fictive score = [ ]).

^j^Proprioceptive score: mean score (number of correct responses) between all the trials of all body parts for the distal propioception task (DPT) (0 = no deficit, 10 = severe deficits).

^k^HAD = hospital anxiety and depression scale.

^l^LOT-r = life orientation test revised.

^m^Defective score for the presence of spatial/personal neglect. n/a = not assessed.

### Lesion mapping

Neuroimaging data were acquired via MRI. We excluded bilateral brain lesions from our analyses (see Supplementary Information). Each patient's lesions were drawn using a semi-automated demarcation,^49,50^ with the ‘Clusterize toolbox’ (https://www.medizin.uni-tuebingen.de/kinder/en/research/neuroimaging/software/) using SPM12 (https://www.fil.ion.ucl.ac.uk/spm/) and Matlab 2018b (https://www.mathworks.com/). Lesion maps and neurological images were subsequently normalized into the common reference space.^51^

The resulting lesion maps were fed to the Lesion Quantification Toolkit^52^ (LQT). This Matlab-based package exploits a brain parcellation into 1034 regions of interest^52^ from MRI data of 1489 typical brains.^53^ For each patient, lesional information was converted into an array describing the percentage of damage of each of these brain parcels (parcel damage). Similarly, LQT exploits tractography data from 841 typical individuals (HCP-842 atlas^54^ to estimate percentage damage severity of 70 macroscale white matter tracts following injury (tract disconnection). LQT also combines grey-matter parcellation and white-matter tractography information to obtain individual connectome-matrices, each describing lesions in terms of pairwise disconnections between grey matter parcels (parcel disconnection).^52^ Parcel damage, tract disconnection and parcel disconnection information were estimated using the default parameters implemented in LQT. Supplementary Fig. 1 displays the overlaps of lesion from our patients. Additional control lesion and network computations were also run (see Supplementary Text and Figs 2–5). Parcel-based and network-based analyses were visualized using MRIcroGL, Surf Ice and DSI Studio. Boxplot visualizations were performed in RStudio.

### Statistical analyses

For behavioural data, statistical analyses were considered two-tailed (*P* < 0.05), and performed using the software SPSS (29.0, https://www.ibm.com/spss). Considering the nature and distribution of data, we used non-parametric tests. We ran Spearman correlation analyses to assess a possible relationship between CD and other variables of interest. Since multiple correlations were tested, Bonferroni correction was applied. To better characterize CD, we compared the severity of CD scores (i.e. the presence of higher scores) in patients with and without neglect using the Mann–Whitney test; we also compared the severity of neurological deficits in patients with and without CD. Finally, we compared the severity of CD scores across the four body parts in patients’ subgroups (knowing and feeling forms of CD) showing disownership for at least two body parts using Friedman analyses of variance by ranks.

Lesion maps and parcel/tract information (from LQT) were modelled as function of symptom severity through linear regression, to identify the neural structures mostly predictive of CD. The analysis was carried out by using VAS differential score (L-R) for the ‘knowing’ (*N* = 100) and the ‘feeling’ (*N* = 67) ownership as dependent variable for each body part, indicating in the model if positive/negative scores are considered defective (see Supplementary Table 2). Region-based analyses were run separately for left and right brain-damaged patients, as these lesions do not overlap with one another and combining them together would have led to an over-conservative minimum overlap parameter (see below). Instead, in network analyses all patients were pulled together, as unilateral lesions can also potentially affect the connectivity with the other hemisphere. Lesional/connectivity information were the main predictor of interest, and age, NIHSS, time since stroke, and lesion size nuisance covariates. Due to the presence of extreme scores (see results), we replaced standard linear models with robust regression,^55-57^ as implemented in the robustfit function from Matlab Statistical Toolbox. The analysis was carried out through a custom Matlab script that repeated the regression in each brain parcel, white-matter tract and pairwise connection. In all cases, we focused only on those parcels/tracts/pairs which were at least 10% damaged in at least 10% of patients (minimum overlap parameter). We considered effects significant if they survived *P* < 0.05 permutation-based correction for multiple comparisons with 2000 permutations of the original dataset.^58^ The number of patients is adequate to investigate the phenomenon. For lesions analyses, a sample size bigger than 90 leads to fully reliable results, even with small effect size.^25^ Parcels surviving permutation corrections were overlaid on a standard Colin 27 template.^59^

## Results

### Behavioural results

#### Prevalence of disownership

Overall, 27 out of 105 (25.7%) patients showed knowing CD, including 17 right-brain, seven left-brain and three bilaterally damaged patients. Among these, 14 (55.5%) presented hand knowing CD (eight for the left hand following a right lesion; six for the right hand following a left lesion). Nine of the knowing CD patients (33.3%) had arm, 12 (44.4%) leg and ten (37%) face disownership.

Furthermore, 14 out of 69 (20.3%) patients in the subgroup tested for the feeling disownership showed feeling CD, including seven right-brain, six left-brain and one bilaterally damaged patients. Among them, eight (57.1%) presented hand feeling CD, eight (57.1%) arm CD, nine (64.3%) leg CD and two (14.3%) face CD.

Four patients with OD were also identified: two (50%) with left-body OD (one right brain-damage with the left hand and face affected and one bilateral with the left arm affected), one (25%) with right-body OD following a left-brain lesion (right hand, arm and face affected) and one (25%) with OD on both hands following a bilateral lesion. All OD patients also showed CD (knowing and feeling) for at least one body part on the VAS.

A clinical picture of one CD patient is reported in Supplementary Table 3.

#### Associated deficits

A small number of cognitive, neurological and upper limb functionality data were missing (see Supplementary Information).

Of the 27 knowing CD patients, 20 (74%) presented with USN, as compared to 29 out of the 78 patients (37%) without knowing CD. 13 of them (48%) also presented with personal neglect, as compared to 13 out of the 78 patients (16.6%) without CD. Of the 14 feeling CD patients, eight (57.1%) also showed USN and five (35.7%) personal neglect (see Table 2). The severity of CD (L-R scores) in patients with and without neglect was comparable (Mann–Whitney, all *P*s > 0.05). Spearman correlation analyses were run to test if CD was related to other deficits. Only one significant correlation survived the correction for multiple comparisons, between feeling CD affecting the hand (L-R scores) and the asymmetric visuo-motor exploration in target cancellation, suggesting that the larger the spatial exploration bias, the worse the feeling of left hand disownership (ρ=−0.37, *P*_bonferroni_ = 0.03) (see Supplementary Table 4 resumes all the correlation analyses results).

#### Neurological severity

Twelve out of 27 knowing CD patients, and nine out of the 14 feeling CD patients presented disownership for at least two body parts, with no significant difference on CD severity across body parts (Friedman analyses, all *P*s > 0.05). With respect to neurological symptoms, a Mann–Whitney non-parametric analysis comparing the neurological severity (NIHSS) in patients with and without CD revealed a significant difference between groups, with medium effect sizes (knowing CD: *U* = 641, *P* = 0.02, *r* = 0.30; feeling CD: *U* = 228, *P* = 0.02, *r* = 0.29): patients exhibiting disownership had a more severe global neurological impairment (mean NIHSS: knowing CD = 4.41; no knowing CD = 1.53; feeling CD = 3.57; no feeling CD = 1.19). A Mann–Whitney non-parametric analysis comparing the motor severity in patients with and without CD revealed a significant difference between groups with medium effect sizes (knowing CD: *U* = 809, *P* = 0.002, *r* = 0.31; feeling CD: *U* = 283.5, *P* = 0.035, *r* = 0.25) suggesting that, on average, CD patients showed a more severe hemiplegia.

### Robust lesion analyses

#### Region-based lesion symptom mapping

**Figure 1:**
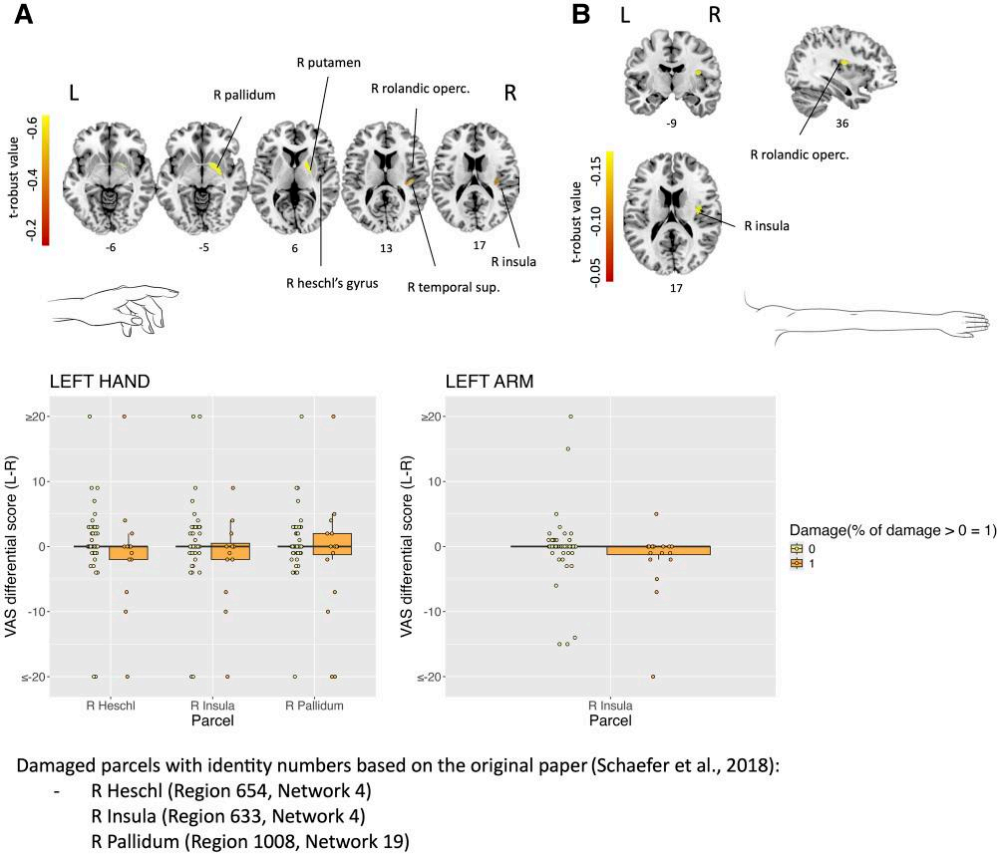
**Robust parcel damage analyses for the ‘knowing’ form of CD.** (**A**) ‘Knowing’ form of CD differential score left (L) minus right (R) in cm for the left hand. (**B**) ‘Knowing’ form of CD differential score left minus right (L-R) in cm for the left arm. Each data point represents the score of a patient. Standard linear models with robust regression were performed (*N* = 100) using 2000 permutations (statistical threshold *P* < 0.05) and took into consideration parcels with at least 10% damaged in at least 10% of the patients’ sample. Age, National Institute of Health Stroke Scale (NIHSS), time since stroke and lesion size were used as covariates.

For the ‘knowing’ manifestation of CD, robust parcel damage analyses revealed significant results for the left upper limb (hand and arm), in which the deficit was associated with damage centred to the right posterior insula (*P* < 0.05). Specifically for the hand, the lesions additionally compromised significantly the right pallidum and the right Heschl's gyrus (*P* < 0.05) (Fig. 1). No significant parcel damage results were observed for the ‘feeling’ of CD.

#### Network-based lesion symptom mapping: structural connectivity

**Figure 2:**
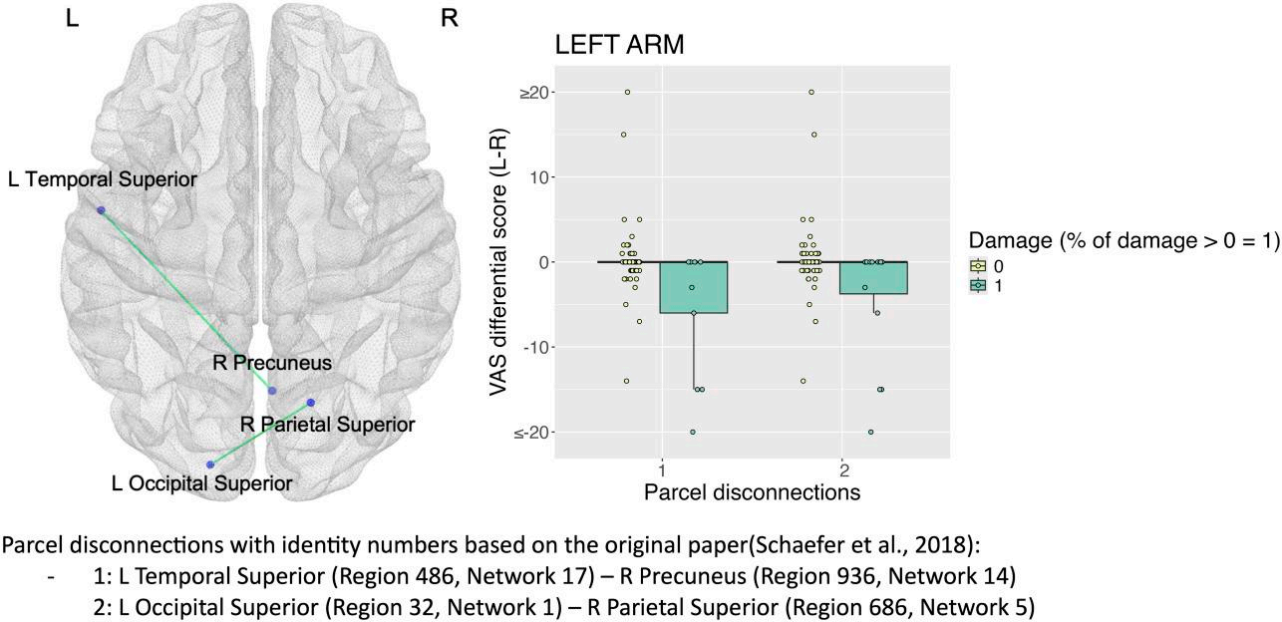
**Robust parcel disconnection analyses for the ‘knowing’ form of CD.** This analysis focuses on CD differential score left (L) minus right (R) in cm for the left arm. Each data point represents the score of a patient. Standard linear models with robust regression were performed (*N* = 100) using 2000 permutations (statistical threshold *P* < 0.05) and took into consideration pairs of parcels with at least 10% damaged in at least 10% of the patients’ sample. Age, National Institute of Health Stroke Scale (NIHSS), time since stroke and lesion size were used as covariates.

Robust parcel disconnection analyses highlighted statistically significant disconnections between the left superior temporal gyrus and the right precuneus, as well as between the left occipital superior gyrus and the right superior parietal gyrus (*P* < 0.05) related to the left arm knowing CD (Fig. 2).

**Figure 3:**
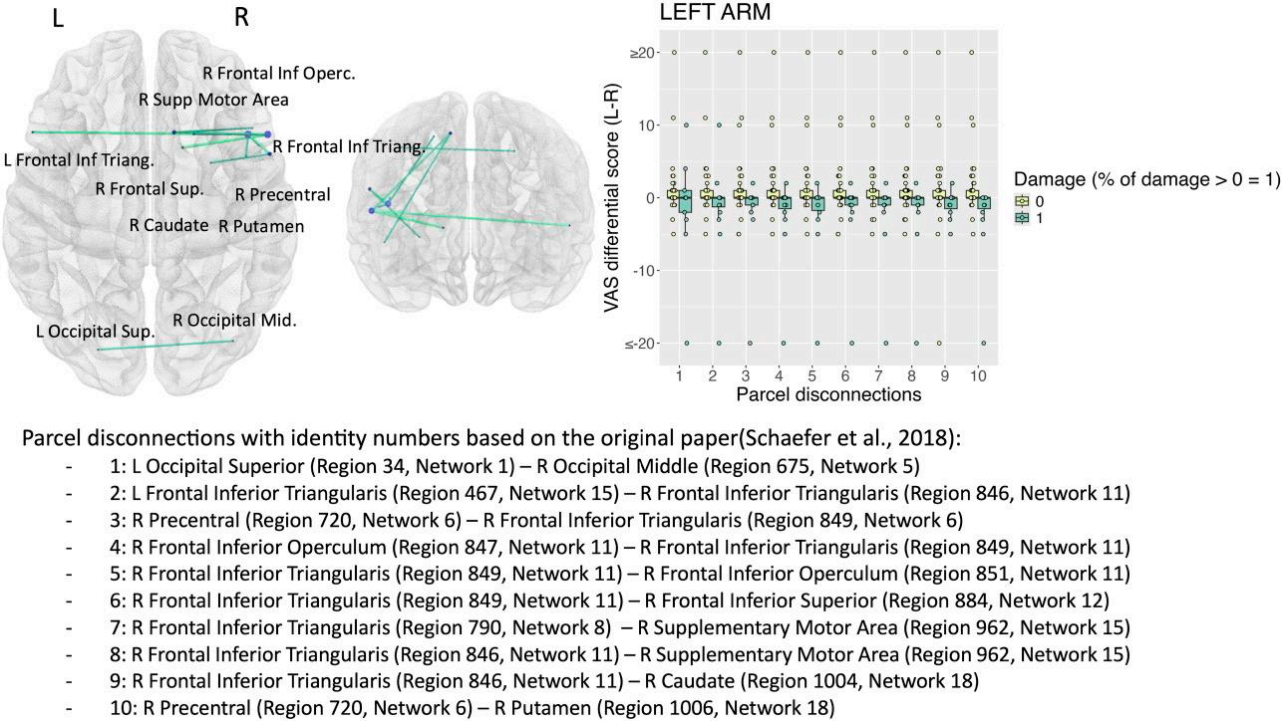
**Robust parcel disconnection analyses for the ‘feeling’ form of CD.** CD differential score left (L) minus right (R) in cm for the left arm. Each data point represents the score of a patient. Standard linear models with robust regression were performed (*N* = 67) using 2000 permutations (statistical threshold *P* < 0.05) and took into consideration pairs of parcels with at least 10% damaged in at least 10% of the patients’ sample. Age, National Institute of Health Stroke Scale (NIHSS), time since stroke and lesion size were used as covariates.

**Figure 4:**
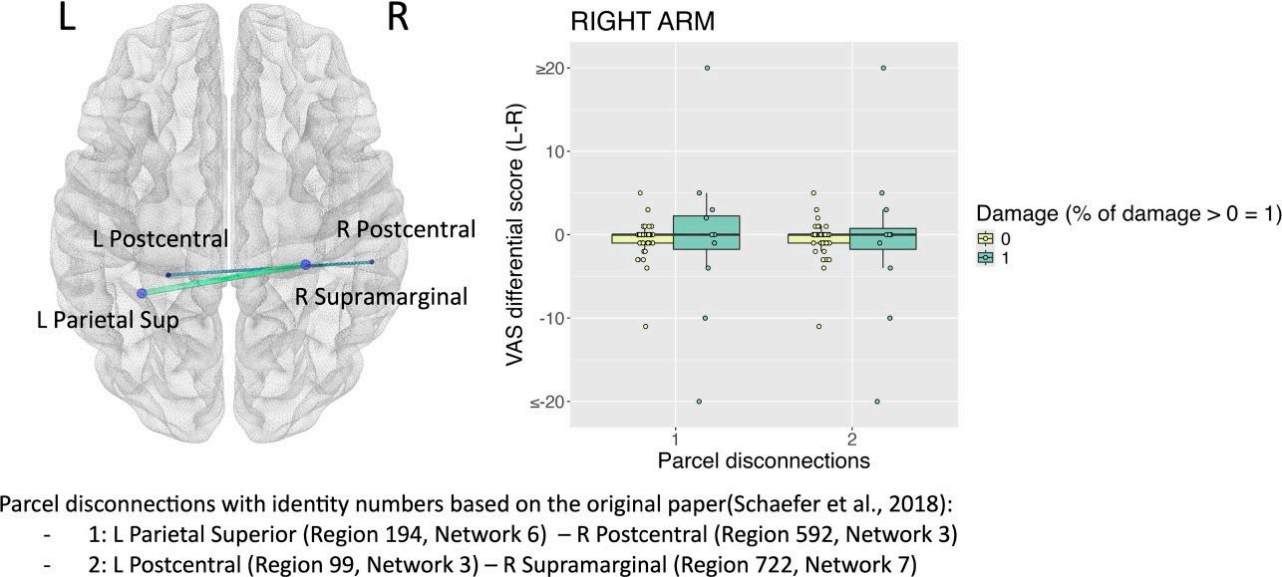
**Robust parcel disconnection analyses for the ‘feeling’ form of CD.** CD differential score left (L) minus right (R) in cm for the right arm. Each data point represents the score of a patient. Standard linear models with robust regression were performed (*N* = 67) using 2000 permutations (statistical threshold *P* < 0.05) and took into consideration pairs of parcels with at least 10% damaged in at least 10% of the patients’ sample. Age, National Institute of Health Stroke Scale (NIHSS), time since stroke and lesion size were used as covariates.

For the feeling CD, we found significant associations of left arm disownership with disconnections between several areas in right frontal lobe, particularly pre-motor cortex, and right subcortical networks, and inter-hemispheric disconnections between occipital parcels (*P* < 0.05) (Fig. 3). Significant disconnections were found between bilateral parietal networks for the right arm feeling CD (*P* < 0.05) (Fig. 4).

**Figure 5:**
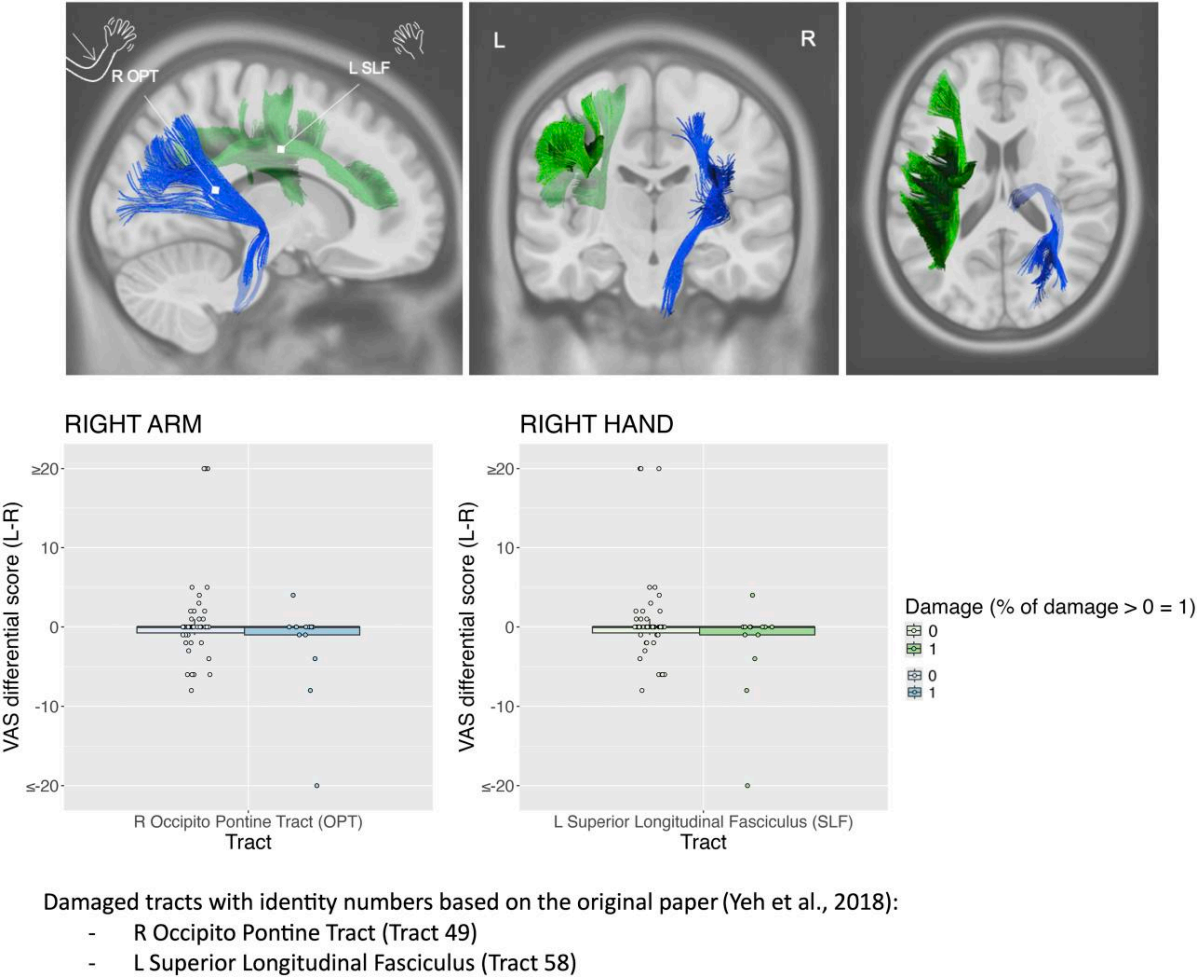
**Robust tract disconnection analyses for the ‘feeling’ form of CD.** Right occipito-pontine tract, R OPT: CD differential score left (L) minus right (R) in cm for the right arm (statistical threshold *P* < 0.01). Left superior longitudinal fasciculus, L SFL: CD differential score (L-R) in cm for the right hand (statistical threshold *P* < 0.03). Each data point represents the score of a patient. Standard linear models with robust regression were performed (*N* = 67) using 2000 permutations and took into consideration tracts with at least 10% damaged in at least 10% of the patients’ sample. Age, National Institute of Health Stroke Scale (NIHSS), time since stroke and lesion size were used as covariates.

Finally, the robust tract disconnection analyses showed no significant results (all *P*s > 0.05) for the knowing CD. For the feeling CD, a significant involvement of the right occipito pontine tract (*P* < 0.01) and the left superior longitudinal fasciculus (*P* < 0.03) was respectively associated with right arm and the right hand CD (Fig. 5).

## Discussion

In this study, combining a neuropsychological approach with state-of-the-art lesion network analyses in an extensive group of patients, we have provided novel insight about the presence of brain networks underlying the sense of humans’ body awareness, and the characterization of hidden forms of disrupted body ownership after a stroke perturbing these systems. Impairments of body ownership may be underestimated in patients, also in the early phase post-disease, highlighting the importance of introducing sensitive, non-verbal response tools. At the neural level, this CD is associated with complex disconnection patterns in bi-hemispheric networks.

### Prevalence and characteristics of covert disownership

Based on a sample of more than 100 patients examined in the first 2 weeks after a stroke, CD was more frequently reported than the OD. This confirms our hypotheses and suggests that OD may recover early (few minutes/hours) following stroke onset, while not explicitly declared impairments can persist. Comparing current data with a previous study,^14^ it seems that the prevalence of CD after right-brain-damage is similar in the early and sub-acute-to-chronic phase. At the clinical level, this covert form can occur without neurological deficits, including proprioceptive ones, which are instead frequently associated with OD.^7^

Validating our predictions, we also report the first evidence that CD can affect right body parts after a left hemispheric lesion, even with a lower frequency (18% versus 8% for knowing CD, 10% versus 8.7% for feeling CD). This underscores a role of the left hemisphere in body parts’ ownership. As previously reported,^7,12^ the use of verbal interview for its assessment might preclude a correct detection of the deficit in patients with aphasia. We highlight the importance of non-verbal tools such as the VAS for the evaluation of disownership, even if not completely free from linguistic processing and, therefore, not applicable in severe aphasic patients. While linguistic deficits are the cognitive disorders more frequently detected after left lesions,^60^ these patients may present some bodily disorders such as pointing to,^61^ naming^62^ and localizing body parts,^63,64^ or planning and executing voluntary actions.^65^ While the specificity of the cognitive profiles of right- versus left-brain-damaged patients has been not explored in our study, we ensured that both left- and right-damaged patients perfectly understood the disownership evaluation, excluding severe aphasic and altitudinal neglect patients.

Even if hand disownership was the more frequent manifestation, patients can experience CD for others specific body parts, including the contralesional hemi-face, a part never explored before in this context. In analogy with other body representation deficits affecting the face, like personal neglect,^66,67^ we demonstrate that unilateral face disownership may be selective in some patients. Moreover, we found no variation in the severity of this disorder across body parts, suggesting that CD may affect body sectors equally.

By analysing the deficits associated with CD, we found a frequent combination with spatial neglect, supporting the view that body ownership is built through a dynamic relationship between body and space.^12,68^ Both the knowing and the feeling forms of CD appear to show a similar clinical pattern, with extra-personal neglect being the most common cognitive associated deficit, instead of personal neglect.^69^ This is in accordance with previous evidence about OD.^9,70-72^ Mancuso *et al*.^73^ described a patient with disownership and delusional attribution of the hand possession, but no signs of personal neglect. Interestingly, Moro *et al*.^69^ analysing an extensive group of patients found that the critical cognitive factor associated with disownership was USN, and not personal neglect (even if assessed with a bodily face, not limb, task). Another deficit not specifically associated with CD in our study is anosognosia. This has been also previously suggested for OD.^69^ Indeed, anosognosia and disownership may stand as two separate processes underlying different neural pathways.^74^ Invernizzi and colleagues explain the frequent co-occurrence of both deficits because of big lesions size. Therefore, the absence of systematic association between anosognosia and CD (see Table 2) may be explained by many patients with subtle neurological and cognitive deficits in our sample. At the neurological level, the severity of sensitive and motor deficits was not consistently associated with CD, even if CD patients presented globally with more severe hemiplegia. Moreover, we did not observe any correlation between arm CD and upper limb functionality, contrary to our expectations (see Limitations).

### Neural systems of covert disownership

We have applied innovative and rigorous methods to unveil the lesion-based and network-based correlates of CD, using robust analyses to consider the extreme values in data distribution. Our results point to the role of specific brain regions and structural networks in both hemispheres in explaining disownership for contralesional body parts. No significant results on the voxel level were found (see Supplementary Information), but for region-based clusters with a functional meaning.

#### ‘Knowing’ body ownership

At the region-based level, analyses revealed the implication of the right insula and basal ganglia in the knowing CD for the left upper limb. In line with our data, these regions have been previously linked to disownership, both for overt and covert forms.^14,22,75^ The insula is a central node in bodily self-consciousness, comprising a fronto-temporo-parietal network, as well as white matter and subcortical grey structures such as the thalamus, basal ganglia and amygdala.^22,24^ Craig^76,77^ attributed to insula a major role in processing internal bodily signals, i.e. interoception. This region is also a polymodal zone implicated in the sense of agency (i.e. subjective awareness of initiating, executing and controlling one's own volitional actions^78^) and ownership,^79^ integrating sensory signals arising from the body with the emotional and motivational context.^80,81^ The insula is highly connected to the limbic system, including the basal ganglia.^82,83^ The latter are thought to partake in cognition and body representation, in addition to their role in motor control and integration, and more generally they constitute key structures for processing afferent and efferent information.^84^ Therefore, both areas provide an important crossing point for integrating information linked to the construction and update of the self, and thus the sense of body awareness.

An important novel finding is that disconnections between right parietal networks and left temporo-occipital areas were also involved in the knowing manifestation of CD for the left arm. These regions have frequently been reported in the literature of disownership.^85-87^ While occipito-temporal areas are recruited in the visual representation of the body,^88-90^ a supramodal body representation is presumably computed in parietal cortex, integrating different sensory modalities.^85^ Therefore, the lack of communication between processing bodily visual features and their integration within a higher-level multimodal body representation may play a key role for the emergence of CD. This is in line with other body representation studies. Bassolino *et al*.^19^ found that chronic stroke patients exhibit a subjective perceived difference in the length and width of the affected limb(s). Moreover, Errante *et al*.^87^ identified the right posterior arcuate fasciculus, a tract disconnecting occipito-temporal and temporo-parietal regions, as critically involved in pathological embodiment (i.e. patients misidentifying other people's limb as their own). A disturbed perceptive processing of the body may therefore constitute an important element for triggering disownership.

In their review, Serino *et al*.^85^ proposed that body ownership emerges from the multisensory integration based on networks in the posterior parietal and the ventral pre-motor cortex. Interestingly, they also proposed that peri-personal space (i.e. the space surrounding the self) shares some brain correlates with body ownership in parietal areas.^24,91^ While peri-personal space defines a space external to the body, its existence is strongly based on the granted ownership and agency, as it is the spatial extent where our own body can interact. Indeed, in patients with body representation disorders, peri-personal space has been shown to be altered for stimuli presented around the impaired body part.^19^ These data support a complex construction of the bodily self not restricted to sensory information conveyed from the body, but also to the continuous update and interaction within the external environment, and thus partially shared brain correlates.

#### ‘Feeling’ body ownership

Changing the semantic structure of the question, we were able to sharpen the detection of hidden forms of CD, targeting the feeling versus the cognitive knowledge of body ownership. Lesion analyses about the feeling of ownership revealed compromised structural tracts and parcels disconnections. Structural disconnections between bilateral occipital parcels and the feeling of left arm disownership, and an impairment of the right occipito-pontine tract for the right arm disownership, were found. This tract, projecting from the occipital cortex to the midbrain, is known to be associated with eye pursuit movement and visual perception.^92^ As also suggested for the knowing form of CD, the visual processing of body parts provides a stable and accurate representation of one's body, essential for tactile processing and movement guidance.^93^ The vision appears an important element contributing to body recognition and action, as well as the visual perspective of the body, participating in the construction, and modulation, of body ownership.^94,95^

We also found extensive disconnections of right bilateral inferior frontal cortex with subcortical basal ganglia networks, pre-motor areas, and contralateral homologous regions for the left arm disownership. Alexander and Crutcher^96^ showed that basal ganglia integrates influences from cortical association and sensorimotor areas and projects to the frontal lobe. Zeller *et al*.^97^ proposed that the pre-motor cortex is not restricted to the movement domain, but also mediates the detection and resolution of potential conflicts in body representation. Thus, these regions may compare the physical perceived body and its internal representation: in case of incongruence, this representation is updated.^97^ The frontal-subcortical network might be implicated in updating this representation to a new condition, but this process may be compromised and fail due to the lesion, possibly resulting into a feeling that the body part is not our own.

Supporting this view, we also found an altered disconnection involving the left superior longitudinal fasciculus for the disownership of the right hand: this tract is known to be involved in motor control and visuo-spatial attention,^98^ disconnecting the ventral pre-motor cortex from the inferior parietal cortex^87^ and supposedly playing a role in multisensory integration. Additionally, our data highlight the role of an altered structural connection between the parietal lobes across the two hemispheres for the right arm disownership. The link between parietal lobes and body processing has been widely reported in the literature.^5,99^ In 1949, Martin Roth^100^ already described a parietal bilateral involvement in body representation disorders manifested by disruptions of praxis and spatial perception functions. Other bodily deficits, such as the feeling of supernumerary limbs, and Xenomelia (i.e. desire for amputation of healthy limbs), have been associated to the right parietal lobe.^101,102^ Studies on autotopagnosia^103^ and heterotopoagnosia,^104^ affecting the ability to point to body parts, suggest that the left parietal lobe is recruited in the self-other discrimination aspect of body representation, and that a lesion of this structure can affect the processing and integration of visual and somatosensory information essential for body identification.^103,105^ More precisely, the location of body parts has been suggested to involve the posterior parietal cortex and the intraparietal sulcus in the left hemisphere.^103,106^ Finally, personal neglect presents with a parietal correlate, involving the right supramarginal, postcentral gyri and the underlying white matter.^107^ An inter-hemispheric integration between the two parietal lobes was considered in a model in which the sense of corporeal identity interacts with the sense of acting on the environment through voluntary motor acts.^108^ According to this model, the right parietal lobe is building a conscious experience of one's body in space from bodily sensory information, while the left parietal lobe may have a distinct capacity to perceive an action as self-generated. Both are coordinated through mechanisms of attention and intention, and contribute to the consciousness of our body in action.^108^ An interruption of such connections may result in a sense of strangeness about body parts, and possibly the feeling of disownership.

Considering these results, the literature and our predictions, the present analyses suggest that both hemispheres play a critical role in building body awareness, differently from the view that body ownership is mainly right-hemispheric based.^7^ This seems specifically the case for the feeling CD, which seems to be more balanced between the right- and left-brain damaged patients than the knowing form, affecting mostly right-brain damaged patients. This is in line with the idea of co-construction of body representation through the concepts of body schema and body image.^109,110^ Indeed, knowing CD seems compatible with the conscious features of body image, which is defined as ‘the long-term spatial properties of one's body’,^111^ while the feeling form may share more communality with the body schema, which is more based on implicit body sensations. Interestingly, the right hemisphere has been proven important for both aspects,^112^ while the body schema would be more left-hemispheric based, as shown in studies about healthy participants and brain-damaged patients.^106,113^ Finally, OD affects the two concepts, with semantic, conscious aspects, as well as some implicit bodily sensation, like proprioception,^7,12^ compromised: in our study, all OD patients also presented with both knowing and feeling disownership. More globally, a meta-analysis of Salvato *et al*.^114^ supports the inter-hemispheric claim and offers valuable clues about how different brain regions might interact for body ownership: occipito-temporal areas play a role in self-recognition, while bilateral parietal regions construct the sense of body representation combining interoceptive and exteroceptive signals. Specifically, the authors point at the role of the left supramarginal gyrus for self-location of body parts in space, and more generally at the role of bilateral supramarginal gyri for the integration of multisensory inputs in the construction or manipulation of body parts ownership.^115^ These multisensory processes should possibly be further integrated in frontal regions, ultimately triggering a unified sense of bodily self.

### Limitations

Some limitations must be considered. First, participants can have different response styles on the VAS (see Ronchi *et al*.^14^). However, because we use a differential score between the left and the right body parts, this should not influence the outcome measures. Moreover, some patients may show scores below the cut-off of healthy participants for body parts on both sides. We decided to rely on the criterium of pathological score affecting the contralesional body part^7^: future studies should explore fluctuations in this score, assessing body parts with multiple trials.

Although we observed CD for the face, its assessment followed a different procedure: indeed, patients do not see their own face when the examiner points at it. A more appropriate evaluation should be conceived, which can result in a higher prevalence of face disownership.

Moreover, at the brain level, we also did not find significant results for the hemi-face. These negative results can be explained by a limited range of pathological scores.

The absence of correlation between CD for the upper limb and upper limb functionality should be taken with caution, because of a possible lack of sensitivity in our measurements. Due to severe motor disorders, upper limb dexterity and grip strength tests could not be assessed in 10 hemiplegic patients, who nevertheless can present severe CD. Being the score based on the differential left minus right arm performances, in hemiplegic patients we could not assess the contralesional arm, losing the differential functionality score. While we applied an imputation method to overcome this limitation, these scores did not reflect the real patients’ performances.

Finally, as a methodological limitation, the reference set^54^ used to build structural connectivity maps is not age-matched with our sample. Nevertheless, this should not affect our results, as age-related changes are more pronounced in the functional than in the structural connectivity measures.^116^ Additionally, the disconnection severity ‘ignores individual anatomical variability’,^52^ partly thanks to the large dataset used for the atlas (*N* > 800), and it has been already used with stroke patients.^52,117^

## Conclusions and future directions

Exploring the wide range of body parts in an extensive sample of post-stroke patients (*N* > 100), and including right and left-brain damage, we have showed that disorders of body ownership are associated with complex structural bilateral networks involved in body perception, update of sensory-motor information, relationship between body and space, and integration of different body representation features.

Further investigations should clarify whether forms of knowing and feeling of ownership exist on a continuum, or rely on distinct mechanisms. It seems that in both cases, an interaction between disrupted high-order cortical networks centred on parieto-insular cortices, and lower ‘unimodal’ brain regions such as sensorimotor cortices,^118^ triggered the experience of disownership. Surprisingly, five patients experiencing the knowing form of CD showed no signs of the feeling form of it. This calls into question the hypothesis of De Vignemont that our judgments of ownership are based on the feeling of ownership.^1^ In their review, Romano *et al*.^42^ defined the amputation-desire as a deficit reflecting the lack of feeling of ownership with a normal acknowledgment of it. Our results show that, when assessed through non-verbal response tools, body representation in stroke patients could reveal the same pattern.

As another future direction, patients with lesions affecting the cerebellum should be included,^119^ considering its strong connection with cerebral areas. Moreover, the association with other self-awareness disorders (e.g. anosognosia) should be deeply explored.

The time course of CD deficits should be also assessed using a follow-up design to compare different phases post-stroke.

Finally, while our study identified the CD using non-verbal, but still explicit, assessment, a fully implicit evaluation may be implemented in the future.

Fine-grained analysis of disownership overtime will help rehabilitation and better predict clinical outcomes. These findings have high relevance not only for clinical populations, but also for exploring the mechanisms of self-consciousness in humans.

## Supplementary Material

fcaf217_Supplementary_Data

## Data Availability

All data are available in the main text, the supplementary materials or in OSF at [https://osf.io/n5xa7/?view_only=1be94ed5a51c4b69975a2b6cd50385ba], reference number [DOI 10.17605/OSF.IO/N5XA7].
