# Supplementary material for: Unveiling covert disownership after stroke: a neuropsychological and neural approach
Source: Brain Commun. 2025 Jun 4;7(3):fcaf217. doi: 10.1093/braincomms/fcaf217 (PMC12204195; doi:10.1093/braincomms/fcaf217)
Supplement: fcaf217_Supplementary_Data [file fcaf217_supplementary_data.pdf]

Supplementary Materials

Supplementary Information

Materials and methods

Neuropsychological evaluation

As a screening, global cognitive efficiency and global executive functions were evaluated through the Montreal Cognitive Assessment[1] (MoCA), and the French version of the Frontal Assessment Battery[2] (FAB). The possible presence of language deficits was assessed with the LAnguage Screening Test[3] (LAST), including oral production and oral comprehension.

An exhaustive evaluation of spatial attention processing was performed using the following tests of the Neglect Evaluation Battery[4] (BEN):

- Extinction to double stimulation for visual, tactile, auditory and motor modalities: four trials for each unimodal and two for each bilateral stimulation.
- Horizontal line bisection: the task's request was to bisect each of the four lines presented on a single A4 paper, centrally; two lines of 5cm and two lines of 10cm were presented.
- Clock drawing: the task's request was to draw the numbers corresponding to the hours in a A4 sheet pre-printed circle, as if it was a clock, in the correct position.
- Overlapping figures task: the task's request was to detect a series of tangled figures, in five subsequent A4 papers; each trial included a central figure (not scored), two on the left and two on the right, for a total of ten left-sided and ten right-sided pictures to be identified.
- Catherine Bergego Scale: it evaluates the degree of difficulty of the patient in ten daily life activities exploring the external space or the body; the statements refer to the presence of lateralized attentional deficits, not neurological (i.e., motor) ones. Two parallel versions of the scale have been administered to the patient and to a therapist, and the discrepancy between the two total scores is indicative of the presence or absence of USN awareness.

The following additional tasks were administered to patients to complete USN evaluation:

- Schenkenberg bisection task[5]: the patient has to bisect 20 lines all printed on a A4 sheet from the top to the bottom; the lines has a length between 10 and 20 cm, and were placed in three different position on the paper (left, center or right).

- Apple cancellation task[6]: the task's request was to cross out all the full apples (N= 50) scattered on a A4 sheet together with distractors (apples open on the left or on the right side).

Moreover, to exclude the presence of altitudinal neglect, which can impact our measure of CD through the vertical VAS, a vertical line bisection was administered using the BEN lines vertically (same number of trials and procedure).

Personal neglect was also assessed by the following two tasks:

- Bisiach modified reaching[7]: the task's request was to reach six different body parts with the ipsilesional hand, eyes open; a score between zero and three for each trial indicated the accuracy, precision and delay in reaching the correct body part.
- Fluff[8] test: the patient has to find some targets placed manually on both contralesional and ipsilesional parts of his/her body with eyes closed.

Finally, we also examined the presence of unawareness following the stroke with well-validated semi-structured interviews[9,10], as follows:

- Anosognosia for illness[9]: the examiner asks the patient the reason of the hospitalization, with questions like "Why are you here? What is the matter with you?". Answers indicating no awareness of the cerebrovascular disease, or confabulations, are considered sign of anosognosia.
- Anosognosia for neurological deficits[10]: the examiner asks the patient if they presented any contralesional difficulties in motor, somato-sensory and/or visual fields functions: a score between 0 (no unawareness) and 3 (complete unawareness even after clinical demonstration) was computed for each neurological function evaluated. This anosognosia was tested only when these functions were affected by the lesions (not assessed in case of unimpaired neurological functions).

Upper limb functional assessment

The following tests have been administered to patients:

- Nine Hole Peg Test: the patient has to remove small pegs one by one from a container, place them into the holes on a board, and put them back into the container as quickly as possible using only the hand being evaluated.

- Jamar hand dynamometer: the patient had to squeeze a dynamometer as tightly as possible, until the cursor stops rising.
- Two-points discrimination: the patient (blindfolded) is asked to discern between one or two points while the tactile stimulation is delivered with progressively decreased distances until an individual threshold is achieved.
- Distal proprioception: the patient was blindfolded and had to report the movement of a specific body part and identify its direction (left, right, up, down; 10 trials for each body part).

Procedure

Selection of patients

Fourteen out of the 119 patients initially tested were excluded for the following reasons, leaving 105 patients in the final sample: two of them dropped out of the protocol, five were excluded due to MRI-related issues (sequelae or missing data), and five showed severe cognitive impairments, including severe linguistic comprehension deficits. Then, based on the previously described assessments, two other patients were excluded based on a deviant bias in the vertical line bisection task, significantly higher than the control participants (see Supplementary information), suggesting the presence of altitudinal neglect.

Associated deficits

From the final sample (N = 105), patients were classified as showing extra-personal spatial and/or personal neglect if they presented with defective, spatially lateralized (left or right) scores in at least one task, based on normative data[4-7,11,12]. For line bisection, the average deviation in millimeters of the patient's mark from the objective midpoint of short and long lines (BEN), or the total percentage of deviation (Schenkenberg), was calculated. For Apple cancellation, L-R egocentric (targets omitted in the left vs. right space) and allocentric (distractors marked with opening on the left vs. right) scores were computed. The Center of Cancellation[12] (CoC) score was also computed and used in subsequent analyses as it provides a robust continuous measure of spatial exploration and extra-personal neglect severity. The clock drawing[4] of the BEN was scored between zero (correct) and two (no digit on one side of the clock). For the overlapping figures task[4] of the BEN, a differential omission score between left and right detected items was

considered. For the Catherine Bergego scale[4], each item was evaluated by the patient or by the therapist between zero and three, depending on the severity of the impairments. Finally, patients were classified as showing personal neglect if they presented with a score above zero on the Bisiach modified reaching[10] and/or a defective lateralized exploration score in the Fluff[8] test, for which we used the improved scoring method of Cocchini and Beschin[11]. The latter was introduced to overcome potential confounding effects (e.g. non spatial deficits) and considers contralesional together with ipsilesional omission scores to the same degree. For upper limb functionality, we used a differential left-right (L-R) score to quantify the difference of the contralesional vs. ipsilesional limb in the following tasks: The Nine hole peg test[13] score was calculated using the number of seconds taken by the patient for each hand (L-R) to complete the test. The Jamar[14] was computed using the measurement in kilograms recorded by the dynamometer for each hand (L-R). The two-points discrimination index[15] recorded the minimal distance in millimeters between the two points where the patient can still report the presence of two stimulations. For the proprioception task[16], the experimenter counted the number of correct items out of 10 trials for each body parts. Ten of the patients presented with severe motor and somato-sensory deficits and were not able to perform the upper limb functional evaluations with the contra-lesional arm: for the Nine hole peg test (N = 10/10), the Jamar test (N = 8/10), the two-points discrimination test (N = 5/10) and the proprioception task (N= 3/10). For the Nine hole peg test and the Jamar, we used an imputation method to replace these missing values. The technique of the maximum or minimum value appeared to us as the most conservative: based on the performance of healthy controls[17,18] we selected the maximum healthy score for the impaired hand and the minimum score for the unimpaired hand and computed a left-right difference (see Table 2). Because we did not find population-based norms with maximum and minimum values for different age ranges related to finger sensitivity, we decided not to apply this procedure to the two-points discrimination test. The proprioception was considered impaired for a specific body part if at least one of the trials was defective (score < 10) for each body part.

Vertical line bisection and patients' selection

The same lines as the horizontal BEN[4] bisection task were used for the testing of altitudinal neglect, including two lines of 5cm and two lines of 20cm. Bisection estimation errors were measured in mm with respect to the center of the line. A cut-off score of was computed based on

the deviation score of 55 healthy age-matched participants (mean (5cm): 0.92 mm, sd: 0.49 mm ; mean (20cm): 7.09 mm, sd: 5.26 mm). We considered a defective altitudinal neglect score a deviation greater or smaller than three standard deviations (SD) from the healthy controls' average scores (cut-off scores (5cm) = min: -0.56 mm, max: 2.40 mm ; (20cm) = min: -8.70 mm, max: 22.88 mm).

Lesion mapping and analyses

We excluded bilateral brain lesions from our analyses. Although the key role of these patients in detecting the clinical prevalence of body disownership, we decided not to include them in the brain analyses. For the region-based analyses, we prevented including the same patients into two groups, as we separated the analyses of patients with right- or left-brain damages. For network-based analyses, as we were interested to explore the presence of whole bilateral brain networks linked to a unilateral deficit caused by a unilateral lesion, bilateral patients would have confounded the role of bilateral network in triggering the unilateral deficits.

In addition to the structural connectivity analysis, we also run a complementary analysis investigating network-based dysfunction from a functional (rather than structural) point of view. Specifically, we modeled lesion data with normative resting-state MRI data, to estimate the overall neural network exhibiting dysfunctional properties following brain injury. This approach followed closely that from previous studies[19,20], and can be subsumed as follows. We analyzed resting-state functional MRI data from N = 94 individuals, matched for age/gender with our clinical sample, which were part of a larger dataset available at OpenfMRI database (accession number ds000221). For each of these individuals, one 15 minutes long resting-state session (see[21] for acquisition details) was analyzed using the SPM12 software (http://www.fil.ion.ucl.ac.uk/spm/), exploiting the preprocessing pipeline from the CONN21a toolbox[22] (https://web.conn-toolbox.org/). Specifically, functional images were realigned to correct for head-movement artefacts and unwrapped to account for geometric distortions related to the magnetic field inhomogeneity. The Artifact (ART) Detection Tools were then used for identification of outlier scans in terms of excessive subject motion and signal intensity spikes. The images were then normalized to a standard stereotaxic space with a voxel-size resolution of 2x2x2mm and smoothed by convolution with an 8mm full-width at half-maximum Gaussian kernel. Following preprocessing, functional signal was denoised through the default pipeline in CONN toolbox to remove components in the

neural signal which were related to (1) white matter and cerebro-spinal fluid signal (first 15 principal components), (2) estimated subject movement parameters (from preprocessing), and (3) the presence of outlier scans (estimated through the ART toolbox during preprocessing). Finally, data were also band-pass filtered (0.008-0.09 Hz) to account for slow-frequency fluctuations (such as scanner drift) and physiological and residual movements artifacts.

The denoised signal was then fed to a connectivity analysis, identifying the brain regions most frequently connected with the lesion location of each patient. The analysis was carried out using the routines for seed-based connectivity implemented in the CONN toolbox, where each lesion mask was specified as a separate seed after having removed those coordinates outside the grey matter[19,23]. Hence, for each of the 100 patients we ran 94 separate general linear models (GLMs) testing in each subject from the associated resting state group those brain regions whose signal was most strongly coupled with time-course extracted from the lesion site. This led to an overall 9400 first-level GLMs (100 patients x 94 resting state subjects). For each neurological patient, the 94 parameter estimates of the associated GLMs were then fed in a second-level one-sample-t-test using random-effect analysis. These were used to create binary network-masks, which included regions which were significantly connected with the seed under voxel-wise threshold corresponding to at least $T \geq 8$[19,23]. These are essentially "extended lesion maps" which include the gray matter portion of lesion site together with the most connected regions and can be easily analyzed through the same processing pipelines for standard voxel-lesion symptom-mapping.

With respect to the analyses, for lesion maps and binary network maps, data were analyzed with standard packages for voxel-based lesion-symptom mapping[24,25] (VLSM, https://aphasialab.org/vlsm2/) modified in such way to replace standard linear models with robust regression. Within this framework, we focused only on coordinates that were implicated in at least 10% of patients. In all analyses, we considered effects significant if survived $p < 0.05$ permutation-based correction for multiple comparisons at the cluster level, with an underlying voxel-level threshold corresponding to $p < 0.001$ (uncorrected).

Results

Behavioural statistical analyses

Chi-square tests were run to test the prevalence of CD between left and right-brain damage. Results show a non-significant difference between left and right-brain damage for the knowing form

(Pearson chi-square= 3.199, two-sided p= .074, φ = 0,18), as well as for the feeling form (Pearson chi-square= 0, two-sided p= 1, φ = 0).

Neurological severity

With respect to the global neurological severity computed with the NIHSS, eight knowing CD and five feeling CD patients presented with motor deficits; nine knowing CD and six feeling CD patients had somatosensory deficits; six knowing CD and two feeling CD patients presented with visual deficits. Unilateral right- and left-brain damaged CD patients showed statistically similar severity scores on the NIHSS scale (knowing CD: U=46, p=.385, r=0.18; feeling CD: U=12.5, p=.405, r=0.24).

Associated deficits

The following data were missing from the final sample. For global neurological and cognitive abilities: N =1 NIHSS; N =1 FAB; N=3 MoCA. For personal neglect: N=1 Fluff test. For unilateral spatial neglect: N=4 Apples test; N=4 Clock; N=1 Bisection 5 cm; N=15 Schenkenberg Bisection; N=1 Catherine Bergego Scale. For questionnaires: N=1 Life Orientation Test-Revised). Moreover, ten patients presented with severe motor and somato-sensory deficits and were not able to perform the functional evaluations with the contra-lesional limbs: for the Nine hole peg test (N=10/10) the Jamar test (N=8/10), the two-points discrimination test (N=5/10) and the proprioception task (N=3/10). Nine hole peg test and Jamar values were replaced with an imputation method (see above). Some additional values were missing for other reasons (e.g. fracture, previous injury): Nine hole peg (N = 15), Jamar (N = 9), two-points discrimination (N = 3) and the proprioception (N = 3) tasks.

Some supplementary analyses have been performed on the sub-group of patients with pure CD (without associated OD) (N= 101). Non-parametric Mann-Whitney analyses comparing the motor score in patients with and without CD showed a significant difference (Mann-Whitney U = 723, p = .009) for the knowing form of CD, but not for the feeling form (Mann-Whitney U = 282.5, p = .124). Comparing the NIHSS score in patients with and without CD, we found a significant difference for the knowing (Mann-Whitney U = 618.5, p = .022) but not for the feeling (Mann-Whitney U = 1175, p = .453) form. Comparing hand CD scores in patients with and without neglect

we showed a non-significant difference (Knowing: Mann-Whitney U = 1141, p = .315; Feeling: Mann-Whitney U = 533, p = .557).

Correlation analyses were run to check if the severity of CD was associated with the severity of USN and personal neglect, the functionality of the upper limb, the medical care delay (time between stroke onset and arrival at the emergency department), the anxiety, depression or dispositional optimism. When analysing the patient with CD without OD, any correlation was significant after correction for multiple comparisons, including the one between the feeling form of CD affecting the hand (L-R scores) and the visuo-motor exploration in the target cancellation.

Voxel-based lesion-symptom mapping analyses

Robust VLSM showed no suprathreshold effect associated with Knowing CD or Feeling CD for all the body parts examined. This result was also demonstrated in the subgroup of CD patients without OD. Despite the absence of significant results at the voxel level, we have found some significant region-based results for the parcels analyses, suggesting that CD seems associated with damage to functionally meaningful clusters (see main manuscript).

Network-based lesion symptom mapping: Structural connectivity

The structural connectivity analyses run on the subgroup of CD patients without OD (N = 66) showed a significant association between the "feeling" manifestation of CD for the left arm ($p<0.05$) and disconnections between several areas in right frontal and basal ganglia (putamen) (see Supplementary Figure 2). These supplementary analyses also revealed significant results for the left arm and the right leg ($p<0.05$), where CD was associated with disconnections in the left superior longitudinal fasciculus (see Supplementary Figure 3).

Network-based lesion symptom mapping: Functional connectivity

Robust functional connectivity analyses run on binary lesion maps showed no significant results for the Knowing as well as for the Feeling of covert disownership in the whole sample of patients. Supplementary robust functional connectivity analyses on CD patients without OD (N = 98) revealed significant results for the "knowing" manifestation of CD for the left arm in which the deficit was associated with dysfunctions centered in a temporo-parieto-occipital network (see Supplementary Figure 4).

Robust functional connectivity analyses in the subgroup of the “feeling” manifestation of CD, without OD patients (N= 66) revealed significant results for the right arm showing an impairment of unilateral left temporo-occipital networks (see Supplementary Figure 5).

This presence of some discrepancies between positive or negative results in structural vs. functional connectivity analyses depending on the sample could be due to the difference in the methodological procedure of these analyses. Functional connectivity takes into consideration the network impairments caused only by the cortical brain region damaged, without the white matter involvement. Moreover, we should consider the non-linear character of functional connectivity, as resting state data inform about which regions are functionally connected, but without information about the presence of direct or indirect connections between the regions. This is in contrast with the structural connectivity analyses we have performed, which computed only direct connections between brain areas.

Supplementary references

1. Nasreddine ZS, Phillips NA, Bédirian V, et al. The Montreal Cognitive Assessment, MoCA: a brief screening tool for mild cognitive impairment. *J Am Geriatr Soc*. Apr 2005;53(4):695-9. doi:10.1111/j.1532-5415.2005.53221.x
2. Dubois B, Slachevsky A, Litvan I, Pillon B. The FAB: a Frontal Assessment Battery at bedside. *Neurology*. Dec 12 2000;55(11):1621-6. doi:10.1212/wnl.55.11.1621
3. Flamand-Roze C, Falissard B, Roze E, et al. Validation of a new language screening tool for patients with acute stroke: the Language Screening Test (LAST). *Stroke*. May 2011;42(5):1224-9. doi:10.1161/strokeaha.110.609503
4. Azouvi P, Samuel C, Louis-Dreyfus A, et al. Sensitivity of clinical and behavioural tests of spatial neglect after right hemisphere stroke. *J Neurol Neurosurg Psychiatry*. Aug 2002;73(2):160-6. doi:10.1136/jnnp.73.2.160
5. Schenkenberg T, Bradford DC, Ajax ET. Line bisection and unilateral visual neglect in patients with neurologic impairment. *Neurology*. May 1980;30(5):509-17. doi:10.1212/wnl.30.5.509
6. Bickerton WL, Samson D, Williamson J, Humphreys GW. Separating forms of neglect using the Apples Test: validation and functional prediction in chronic and acute stroke. *Neuropsychology*. Sep 2011;25(5):567-80. doi:10.1037/a0023501
7. Fortis P, Maravita A, Gallucci M, et al. Rehabilitating patients with left spatial neglect by prism exposure during a visuomotor activity. *Neuropsychology*. Nov 2010;24(6):681-97. doi:10.1037/a0019476
8. Cocchini G, Beschin N, Jehkonen M. The Fluff Test: A simple task to assess body representation neglect. *Neuropsychological Rehabilitation*. 2001;11(1):17-31.
9. Cutting J. Study of anosognosia. *Journal of Neurology, Neurosurgery & Psychiatry*. 1978;41(6):548-555.
10. Bisiach E, Vallar G, Perani D, Papagno C, Berti A. Unawareness of disease following lesions of the right hemisphere: anosognosia for hemiplegia and anosognosia for hemianopia. *Neuropsychologia*. 1986;24(4):471-82. doi:10.1016/0028-3932(86)90092-8
11. Cocchini G, Beschin N. The Fluff test: Improved scoring system to account for different degrees of contralesional and ipsilesional personal neglect in brain damaged patients. *Neuropsychol Rehabil*. Jan 2022;32(1):69-83. doi:10.1080/09602011.2020.1797828
12. Rorden C, Karnath HO. A simple measure of neglect severity. *Neuropsychologia*. Jul 2010;48(9):2758-63. doi:10.1016/j.neuropsychologia.2010.04.018
13. Kellor M, Frost J, Silberberg N, Iversen I, Cummings R. Hand strength and dexterity. *The American journal of occupational therapy: official publication of the American Occupational Therapy Association*. 1971;25(2):77-83.
14. Schmidt RT, Toews JV. Grip strength as measured by the Jamar dynamometer. *Arch Phys Med Rehabil*. Jun 1970;51(6):321-7.
15. Weber E. Ueber den tastsinn. *Arch Anat Physiol wiss Med Berlin*. 1835;1:152-159.
16. Richardson JK. The clinical identification of peripheral neuropathy among older persons. *Arch Phys Med Rehabil*. Nov 2002;83(11):1553-8. doi:10.1053/apmr.2002.35656
17. Wang YC, Bohannon RW, Kapellusch J, Garg A, Gershon RC. Dexterity as measured with the 9-Hole Peg Test (9-HPT) across the age span. *J Hand Ther*. Jan-Mar 2015;28(1):53-9; quiz 60. doi:10.1016/j.jht.2014.09.002

18. Werle S, Goldhahn J, Drerup S, Simmen BR, Sprott H, Herren DB. Age- and gender-specific normative data of grip and pinch strength in a healthy adult Swiss population. *J Hand Surg Eur Vol*. Feb 2009;34(1):76-84. doi:10.1177/1753193408096763
19. Corradi-Dell'Acqua C, Ronchi R, Thomasson M, Bernati T, Saj A, Vuilleumier P. Deficits in cognitive and affective theory of mind relate to dissociated lesion patterns in prefrontal and insular cortex. *Cortex*. Jul 2020;128:218-233. doi:10.1016/j.cortex.2020.03.019
20. Joutsa J, Corp DT, Fox MD. Lesion network mapping for symptom localization: recent developments and future directions. *Curr Opin Neurol*. Aug 1 2022;35(4):453-459. doi:10.1097/wco.0000000000001085
21. Babayan A, Erbey M, Kumral D, et al. A mind-brain-body dataset of MRI, EEG, cognition, emotion, and peripheral physiology in young and old adults. *Sci Data*. Feb 12 2019;6:180308. doi:10.1038/sdata.2018.308
22. Nieto-Castanon A. *Handbook of functional connectivity magnetic resonance imaging methods in CONN*. Hilbert Press; 2020.
23. Wawrzyniak M, Klingbeil J, Zeller D, Saur D, Classen J. The neuronal network involved in self-attribution of an artificial hand: A lesion network-symptom-mapping study. *Neuroimage*. Feb 1 2018;166:317-324. doi:10.1016/j.neuroimage.2017.11.011
24. Bates E, Wilson SM, Saygin AP, et al. Voxel-based lesion-symptom mapping. *Nat Neurosci*. May 2003;6(5):448-50. doi:10.1038/nn1050
25. Wilson SM, Henry ML, Besbris M, et al. Connected speech production in three variants of primary progressive aphasia. *Brain*. Jul 2010;133(Pt 7):2069-88. doi:10.1093/brain/awq129

Supplementary figures

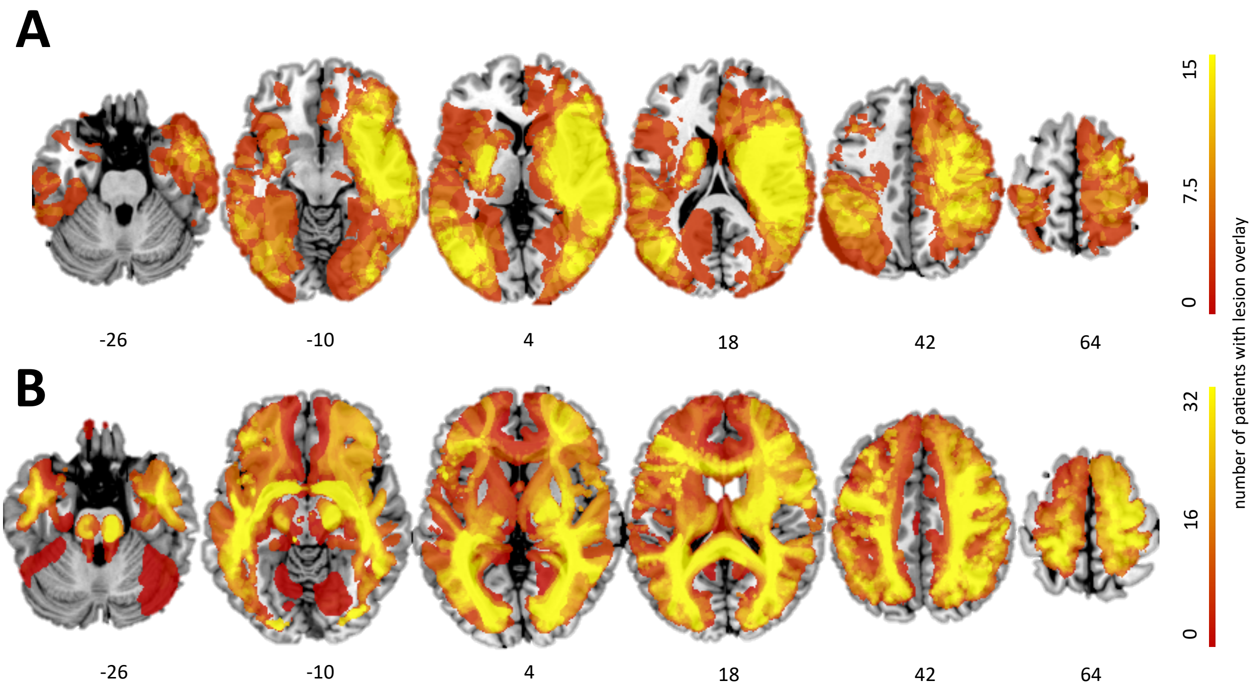

Supplementary Fig.1 Overlaps of the lesion and network maps from our patients (*N* = 100).

(**A**) Raw lesion maps (**B**) Structural Connectivity maps.

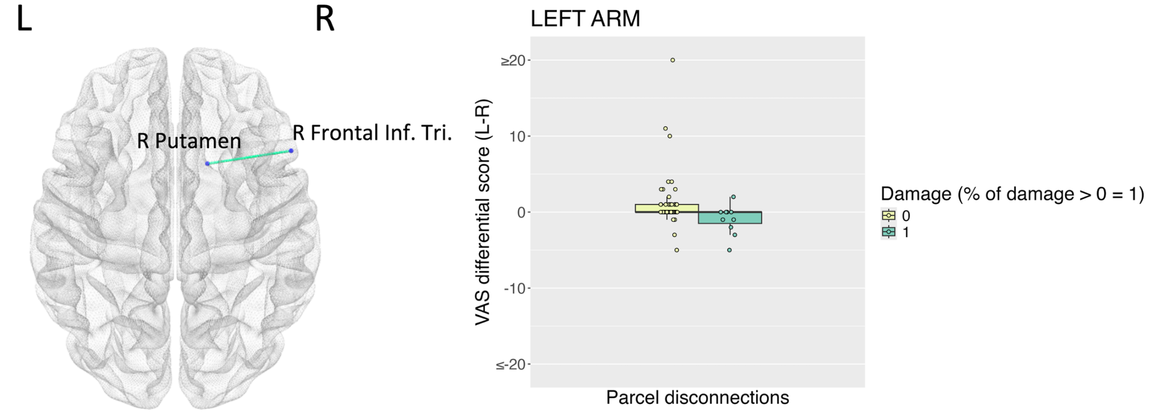

Parcel disconnection with identity numbers based on the original paper(Schaefer et al., 2018):

- R Putamen (Region 1004, Network 18) – R Frontal Inferior Triangularis (Region 846, Network 11)

Supplementary Fig. 2 Robust parcel disconnection analyses for the "feeling" form of covert disownership (CD) without OD patients.

CD differential score left minus right (L-R) in cm for the left arm. Analyses were performed (N = 66) using 2000 permutations (statistical threshold $p < 0.05$) and took into consideration pairs of parcels with at least 10% damaged in at least 10% of the patients' sample. Each data point represents the score of a patient. Age, National Institute of Health Stroke Scale (NIHSS), time since stroke, and lesion size were used as covariates.

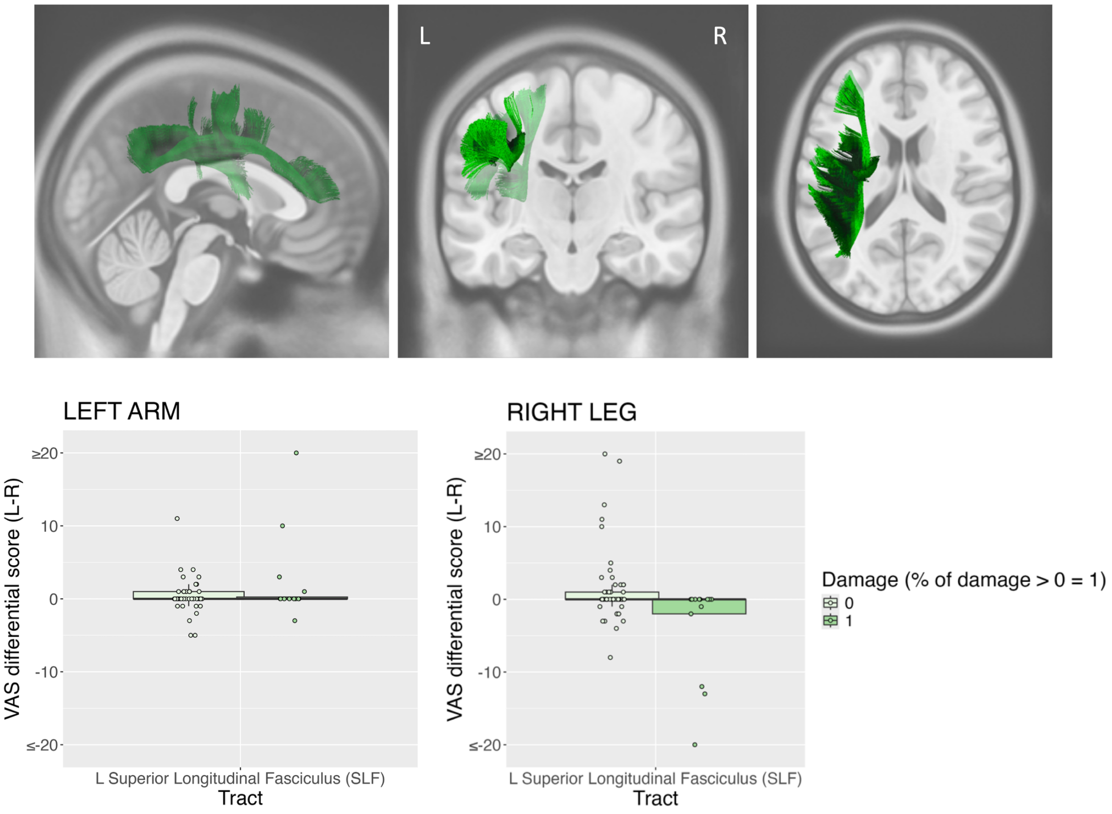

Damaged tract with identity number based on the original paper(Schaefer et al., 2018):

- L Superior Longitudinal Fasciculus (Tract 58)

Supplementary Fig. 3 Robust tract disconnection analyses for the "feeling" form of covert disownership (CD) without OD patients.

CD differential score left minus right (L-R) in cm for the left arm and right leg. Analyses were performed (N = 66) using 2000 permutations (statistical threshold $p < 0.05$) and took into consideration pairs of parcels with at least 10% damaged in at least 10% of the patients' sample. Each data point represents the score of a patient. Age, National Institute of Health Stroke Scale (NIHSS), time since stroke, and lesion size were used as covariates.

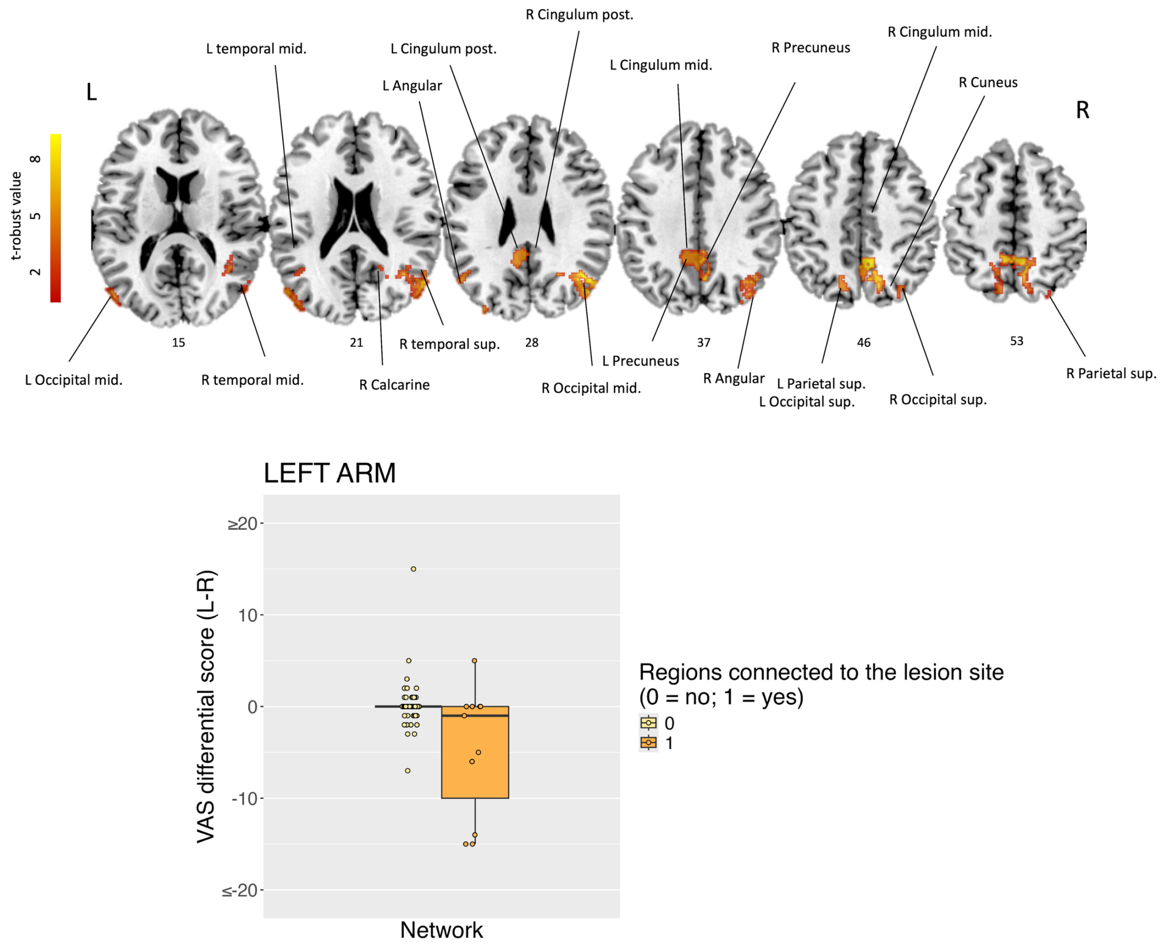

Supplementary Fig.4 Robust lesion network mapping analyses for the "knowing" form of covert disownership (CD) without OD patients.

CD differential score left minus right (L-R) in cm for the left arm. Analyses were performed (*N* = 98) using 2000 permutations (statistical threshold $p < 0.001$) and took into consideration pairs of parcels with at least 10% damaged in at least 10% of the patients' sample. Each data point represents the score of a patient. Age, National Institute of Health Stroke Scale (NIHSS), time since stroke, and lesion size were used as covariates.

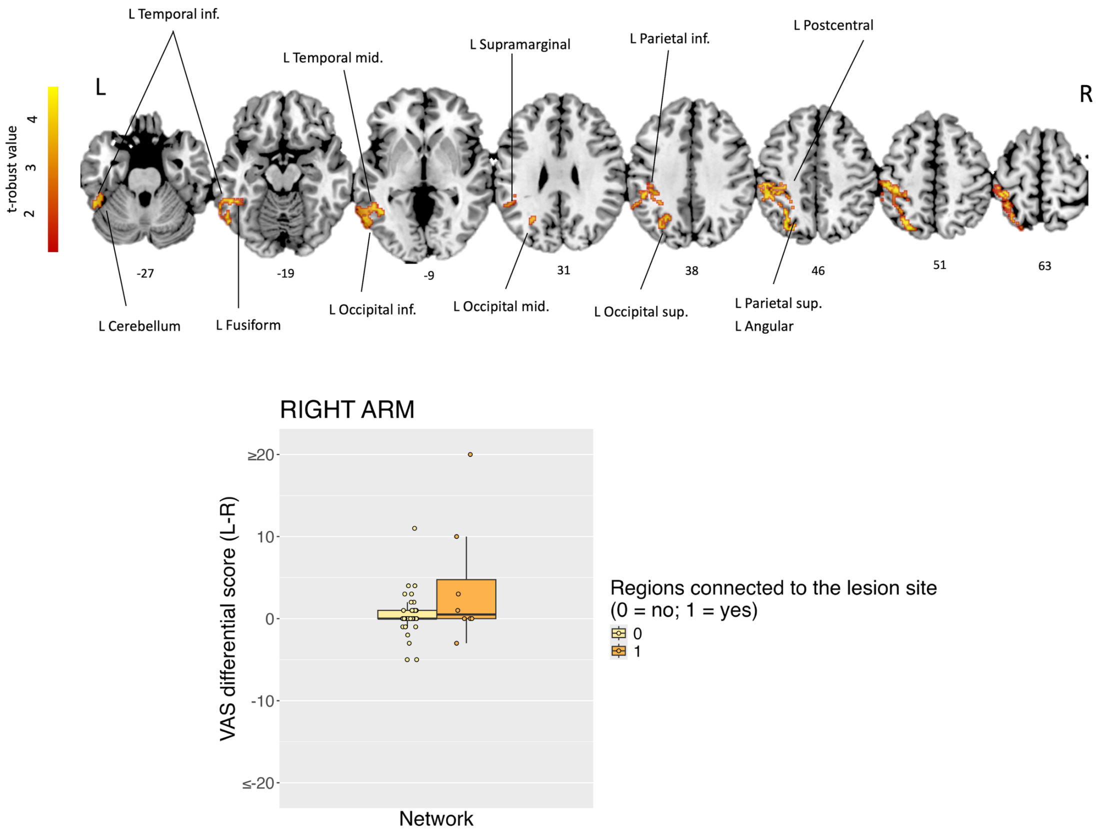

Supplementary Fig.5 Robust voxel lesion network mapping analyses for the "feeling" form of covert disownership (CD) without OD patients.

CD differential score left minus right (L-R) in cm for the right arm. Analyses were performed (*N* = 66) using 2000 permutations (statistical threshold $p < 0.001$) and took into consideration pairs of parcels with at least 10% damaged in at least 10% of the patients' sample. Each data point represents the score of a patient. Age, National Institute of Health Stroke Scale (NIHSS), time since stroke, and lesion size were used as covariates.

Supplementary tables

Supplementary Table 1 Cut-off scores used for the classification of OD[a], Knowing CD[b], and Feeling CD[b] for each body part

DISOWN	Left Hand	Right Hand	L-R[c]	Left Arm	Right Arm	L-R[c]	Left Leg	Right Leg	L-R[c]	Left Face	Right Face	L-R[c]
**OD[a] (0-4)**	< 4	< 4		< 4	< 4		< 4	< 4		< 4	< 4	
**Knowing CD[b] (cm)**	20.58	20.66	±0.24[b] (mean[e] = -0.011, sd[f] = 0.078)	20.61	20.61	±0.17[b] (mean[e] = 0, sd[f] = 0.058)	20.7	20.6	±0.14[b] (mean[e] = 0.006, sd[f] = 0.050)	20.66	20.59	±0.18[b] (mean[e] = 0.006, sd[f] = 0.063)
**Feeling CD[b] (cm)**	20.12	20.12	±0.45[b] (mean[e] = -0.009, sd[f] = 0.147)	20.18	20.14	±0.24[b] (mean[e] = 0.013, sd[f] = 0.086)	20.13	20.14	±0.53[b] (mean[e] = 0, sd[f] = 0.178)	18.67	19.27	±0.77[b] (mean[e] = -0.027, sd[f] = 0.249)

[a] OD = Overt Disownership
[b] CD = Covert Disownership
[c] L-R = score left-right.
[d] ± = L-R score can be > x or < -x.
[e] mean = left minus right mean differential score of the controls (N = 55) on the VAS scale
[f] sd = standard deviation of the control's performance

Supplementary Table 2: Summary of the robust lesion analyses inputs

**Analyses**	**Voxel-based lesion symptom mapping**[a]		**Network based lesion symptom mapping : structural connectivity**[b]		**Network based lesion symptom mapping : functional connectivity**[c]	
**Groups**	**Left body part CD**[d]	**Left body part CD**[d]	**Left body part CD**[d]	**Right body part CD**[d]	**Left body part CD**[d]	**Right body part CD**[d]
**Dependent variable**	VAS differential score L-R[e] for<br>- The hand<br>- The arm<br>- The leg<br>- The face		VAS differential score L-R[e] for<br>- The hand<br>- The arm<br>- The leg<br>- The face	VAS differential score R-L[e] for<br>- The hand<br>- The arm<br>- The leg<br>- The face	VAS differential score L-R[e] for<br>- The hand<br>- The arm<br>- The leg<br>- The face<br>(Low score= defective score)	VAS differential score L-R[e] for<br>- The hand<br>- The arm<br>- The leg<br>- The face<br>(High score = defective score)

[a] For voxel-based lesion analyses, the computation were ran separately for right vs. left lesions, and for the Knowing VAS differential scores vs. the Feeling VAS differential scores.
[b] For structural connectivity, right and left lesions were processed at the same time, identifying the side of the deficit using the VAS differential L-R vs. R-L. The Knowing VAS differential scores vs. the Feeling VAS differential scores were ran separately.
[c] For functional connectivity, right and left lesions were processed at the same time, identifying the side of the deficit using a parameter to indicate the side of the impairment.
[d] CD = CD scores
[e] L-R = VAS differential score L-R (score left-right).

Supplementary Table 3 Case report of S.T., a patient[a] showing signs of right covert disownership

**Lesion**	Left acute stroke lesion (corona radiata)
**Neurological exam**	Ataxia (right upper and lower limbs)
**Neuropsychological exam :**	
○ **Anosognosia**	Anosognosia for illness and related dysfunctions (Starkstein and Bisiach's score)
○ **Spatial deficits**	Right body personal neglect (Fluff Test) and allocentric spatial neglect (Apples Test)
○ **Linguistic deficits**	None
○ **Questionnaires**	Moderate anxiety and depression scores
**Upper Limb Functionality**	Fine sensory and fingers dexterity
**Overt Disownership**	Delayed and uncertain responses for the right hand, arm and face
**Covert Disownership**	Right hand, arm and face attributed to others (e.g. "this arm belongs to my grandchild"). When asked, she says that it is not supposed to, and she expresses a feeling of discomfort.

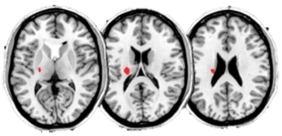

[a] Female, Age: 69 y.o., Right-handed. Assessment two days post-stroke.

Supplementary Table 4 Significant spearman correlation analyses between CD[a] scores[b] and associated deficits

	NIHSS[c]	CoC[d]	BEN bisection (5cm)	BEN bisection (20cm)	Schenkenberg	Jamar	NHPT[e]
**Knowing CD[a]**							
**Arm**	$p_{bonferroni} = 0.13$						
**Leg**						$p_{bonferroni} = 0.13$	
**Face**			$p_{bonferroni} = 0.26$				
**Feeling CD[a]**							
**Hand**		$p_{bonferroni} = 0.03$*		$p_{bonferroni} = 0.39$	$p_{bonferroni} = 0.39$		
**Arm**			$p_{bonferroni} = 0.39$				
**Leg**							$p_{bonferroni} = 0.39$

[a] CD = Covert Disownership
[b] CD scores = VAS differential scores left-right.
[c] NIHSS = National Institute of Health Stroke Scale.
[d] CoC = Center of Cancellation (Apples test).
[e] NHPT = Nine-hole peg test, Jamar (score left-right).
* = significative results, surviving to Bonferroni correction
